# WRKY1 represses the WHIRLY1 transcription factor to positively regulate plant defense against geminivirus infection

**DOI:** 10.1371/journal.ppat.1011319

**Published:** 2023-04-07

**Authors:** Shaoshuang Sun, Shupeng Li, Xueping Zhou, Xiuling Yang

**Affiliations:** 1 State Key Laboratory for Biology of Plant Diseases and Insect Pests, Institute of Plant Protection, Chinese Academy of Agricultural Sciences, Beijing, China; 2 State Key Laboratory of Rice Biology, Institute of Biotechnology, Zhejiang University, Hangzhou, China; The Ohio State University, UNITED STATES

## Abstract

Geminiviruses constitute the largest group of known plant viruses and cause devastating diseases and economic losses in many crops worldwide. Due to limited naturally occurring resistance genes, understanding plant antiviral defense against geminiviruses is critical for finding host factors of geminiviruses and development of strategies for geminivirus control. Here we identified NbWRKY1 as a positive regulator of plant defense against geminivirus infection. Using tomato yellow leaf curl China virus/tomato yellow leaf curl China betasatellite (TYLCCNV/TYLCCNB) as a representative geminivirus, we found that NbWRKY1 was upregulated in response to TYLCCNV/TYLCCNB infection. Overexpression of NbWRKY1 attenuated TYLCCNV/TYLCCNB infection, whereas knockdown of NbWRKY1 enhanced plant susceptibility to TYLCCNV/TYLCCNB. We further revealed that NbWRKY1 bound to the promoter of the NbWHIRLY1 (NbWhy1) transcription factor and inhibited the transcription of NbWhy1. Consistently, NbWhy1 negatively regulates plant response against TYLCCNV/TYLCCNB. Overexpression of NbWhy1 significantly accelerated TYLCCNV/TYLCCNB infection. Conversely, knockdown of NbWhy1 led to impaired geminivirus infection. Furthermore, we demonstrated that NbWhy1 interfered with the antiviral RNAi defense and disrupted the interaction between calmodulin 3 and calmodulin-binding transcription activator-3. Moreover, the NbWRKY1-NbWhy1 also confers plant antiviral response toward tomato yellow leaf curl virus infection. Taken together, our findings suggest that NbWRKY1 positively regulates plant defense to geminivirus infection by repressing NbWhy1. We propose that the NbWRKY1-NbWhy1 cascade could be further employed to control geminiviruses.

## Author summary

Plants employ multiple layers of antiviral defense to fight against geminivirus infection. Transcription factors are key masters that can reprogram transcription and enable plant to respond to different stimuli adequately and flexibly. Here we explore the molecular basis of the NbWRKY1 transcription factor in regulating geminivirus infection. We provide evidence that NbWRKY1 positively regulates plant defense response against geminivirus. We also identify that NbWRKY1 binds to the promoter of the NbWHIRLY1 (NbWhy1) transcription factor and represses the transcription of *NbWhy1*. Furthermore, we show that NbWhy1 is a negative regulator of antiviral RNAi defense and interferes with the interaction of calmodulin 3 and calmodulin-binding transcription activator-3 to promote geminivirus infection. Collectively, this study reveals that NbWRKY1 represses NbWhy1 to positively regulate plant defense to geminivirus infection. WHIRLY transcription factors have been previously described to a positive regulatory role in basal and induced defense responses in *Arabidopsis*, but their roles in plant virus infection remains exclusive. Our study demonstrates a pro-viral role of NbWhy1 in geminivirus infection, expanding our knowledge of the NbWRKY1-NbWhy1 cascade during plant-geminivirus interaction.

## Introduction

Plants are continuously exposed to abiotic and biotic interactions and have empowered sophisticated immune systems to fight against unfavorable stresses. Transcription factors are important regulators of signal transduction that reprogram transcription and enable plant to respond to different stimuli adequately and flexibly [1,2]. WRKY proteins comprise a large family of transcription factor that are known for the conserved WRKY domain (WRKYGQK) in the DNA binding domain of the N-terminus and a zinc finger-like motif in the C-terminus [3]. Although the number of WRKY members in different plant species varied, WRKYs are classified into three major groups based on the number of WRKY domain and the feature of their zinc finger-like motif. Group I WRKY proteins contain two WRKY domains and two C2H2 zinc-finger motifs, group II WRKY proteins comprise one WRKY domain and have a C2H2 zinc-finger motif, and group III WRKY proteins consist of one WRKY domain and a C2HC zinc-finger motif [4]. Group II WRKY proteins can be further divided into five subgroups (IIa, IIb, IIc, IId, and IIe) based on additional conserved structural motifs [5,6]. Several WRKY proteins have been proven to participate in pathogen-associated molecular pattern (PAMP)-triggered immunity (PTI) and effector-triggered immunity (ETI) [7,8]. WRKY directly or indirectly interacts with PAMPs/effector proteins or modulates mitogen-activated protein kinases to inhibit or activate plant defense responses [9]. Different WRKYs play positive or negative roles in regulating plant defense against diverse bacterial and fungal pathogens. OsWRKY51 renders rice resistance to *Xanthomonas oryzae* pv. oryzae (Xoo) by activation of the expression of *OsPR10a* and *OsWRKY10*, whereas *Arabidopsis* WRKY33 enhanced the susceptibility to *Botrytis cinerea* and *Alternaria brassicicola* [10–12]. Involvement of WRKY transcription factor in defending against plant virus infection has also been reported. For example, WRKY1-WRKY3 contributed to the *N* gene-mediated resistance to tobacco mosaic virus (TMV) in *Nicotiana benthamiana* plants (Liu *et al*. 2004), while WRKY8 functions in antiviral response against crucifer-infecting TMV (TMV-cg) by restricting the long-distance movement of TMV-cg in *Arabidopsis* [13]. WRKY has also been implicated in plant-vector-geminivirus interaction, and the βC1 protein encoded by begomovirus-associated betasatellite interacts with and hijacks AtWRKY20 to benefit whitefly vectors but deters two nonvector competitors [14]. Due to the structure of the WRKY domain, WRKY can bind to the typical W-box (TTGAC[T/C]) cis-elements that are often found in the promoters of putative downstream target genes to affect their transcription [15]. WRKYs also regulate plant signaling through physical interaction with proteins involved in transcription, signaling, plant defense, and other cellular processes [8,16]. However, little is known about the transcriptional cascade of WRKY transcription factor in plant virus infection.

Geminiviruses are a group of circular single-stranded DNA (ssDNA) viruses that package their ssDNA genome(s) in twinned icosahedral virions. They comprise the largest and more diverse genera of plant viruses and cause devastating diseases and significant yield losses in many economically important crops globally [17]. 520 species of geminiviruses have been identified and assigned to 14 genera based on the pairwise sequence similarity, genomic structure, transmission vector, and host range (*Becurtovirus*, *Begomovirus*, *Capulavirus*, *Citlodavirus*, *Curtovirus*, *Eragrovirus*, *Grablovirus*, *Maldovirus*, *Mastrevirus*, *Mulcrilevirus*, *Opunvirus*, *Topilevirus*, *Topocuvirus*, and *Turncurtovirus*) [18]. Begomoviruses comprise the largest and diverse genus of the family *Geminiviridae* and have either monopartite or bipartite ssDNA component(s) of less than 3 kb each. Bipartite begomoviruses contain two DNA components designated as DNA-A and DNA-B. Monopartite begomoviruses have a single DNA component that contains six open reading frames, namely C1, C2, C3, and C4 on the complementary strand, and V1 and V2 on the virion strand. Some monopartite begomoviruses are frequently associated with a betasatellite that can enhance the virulence of their helper viruses [19]. As geminiviruses rely on their multi-functional proteins to tailor the environment of plant cell to facilitate their infection, geminiviral proteins are thus excellent molecular weapons that can be used to unravel the secrets of the antiviral arsenal of plants [20]. Such a good example is the βC1 protein encoded by geminivirus-associated betasatellite, which function in different modes of action to counteract antiviral defenses that manifest at genomic (i.e., DNA methylation), transcriptional (i.e., transcriptional reprogramming and histone modification), and post-translational levels (i.e., post-translational modification and autophagy) [21,22].

RNA silencing is a well-established antiviral immunity system in plants [23,24]. The core components, such as Dicer-like endoribonucleases, Argonautes, double-stranded RNA-binding proteins, and RNA-dependent RNA polymerases coordinate to target viral RNA for degradation or guide methylation of viral DNA to limit virus infection [25,26]. Previously, several RNAi genes were reported to be post-transcriptionally regulated, however, how RNAi genes are transcriptionally controlled during plant virus infection remained elusive until a recent study by Wang et al [27]. By using the V2 protein of cotton leaf curl Multan virus as bait, they have identified a V2-interacting calmodulin (CaM) and revealed that a CaM-dependent transcription factor-3 (CAMTA3) mediates the genetic link between calcium signaling and antiviral RNA. They proposed a model that Ca^2+^-CaM-CAMTA3-BN2/RDR6 signaling cascade primes antiviral RNAi defense and V2 interferes with RNAi by disrupting the CaM-CAMTA3 interaction [27]. Given that transcription factors enable plants to respond flexibly and timely, we hypothesized a different combinational interaction between transcription factor and antiviral defense during geminivirus infection.

Our recent study has shown that NbWRKY1, a Group I WRKY transcription factor, positively regulates the incompatible interaction between a mulcrilevirus, mulberry mosaic dwarf associated virus (MMDaV) and *N*. *benthamiana* [28]. However, little is known about the role of NbWRKY1 in compatible plant-geminivirus interaction and the transcriptional regulatory cascade of NbWRKY1 in geminivirus infection. In this study, we investigated whether NbWRKY1 is involved in compatible plant-geminivirus interaction and, if so, how NbWRKY1 responds to geminivirus infection. Using tomato yellow leaf curl China virus/tomato yellow leaf curl China betasatellite (TYLCCNV/TYLCCNB), the representative begomovirus-betasatellite disease complex in China as an example, we showed that NbWRKY1 was upregulated upon TYLCCNV/TYLCCNB infection and positively regulated plant defense to TYLCCNB/TYLCCNB infection. We demonstrated that NbWRKY1 bound to the promoter of NbWHIRLY1 (NbWhy1) and repressed the transcription of *NbWhy1*, a negative regulator of RNAi that facilitated TYLCCNV/TYLCCNB infection. Moreover, the NbWRKY1-NbWhy1 module positively regulated plant antiviral response to another monopartite begomovirus, tomato yellow leaf curl virus (TYLCV). Taken together, our results indicate that NbWRKY1 positively regulates plant defense against geminivirus infection by repressing NbWhy1.

## Results

### NbWRKY1 is stimulated upon TYLCCNV/TYLCCNB infection

**Fig 1:**
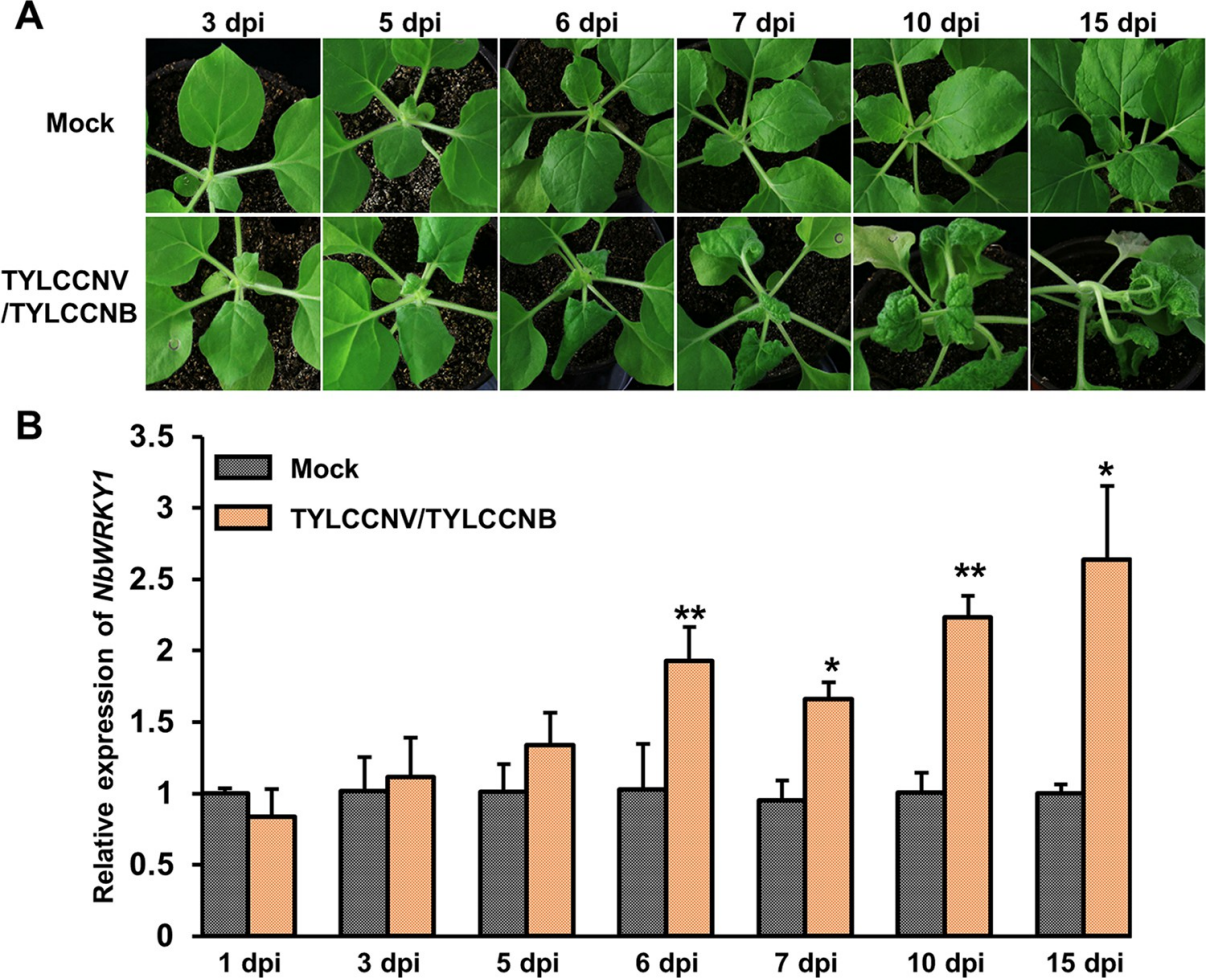
NbWRKY1 is upregulated in response to tomato yellow leaf curl China virus/tomato yellow leaf curl China betasatellite (TYLCCNV/TYLCCNB) infection. (A) Symptoms of *Nicotiana benthamiana* plants agroinoculated with the pBinPLUS vector or the infectious clone of TYLCCNV and TYLCCNB. Symptoms were monitored at various days post inoculation (dpi). (B) Quantitative real-time (qRT-PCR) analysis of *NbWRKY1* mRNA level in plants as indicated in (A). *NbGAPDH* was used as an internal control. Mean and standard deviation of three independent plants are shown. Double and single asterisks indicate significant statistical differences between two treatments at *p*<0.01 and *p*<0.05 based on Student’s *t* test, respectively.

To investigate whether NbWRKY1 is involved in TYLCCNV/TYLCCNB infection, we inoculated the infectious clone of TYLCCNV/TYLCCNB into *N*. *benthamiana* via *Agrobacterium*-mediated infiltration. The disease symptoms of inoculated *N*. *benthamiana* plants were monitored and the expression of the *NbWRKY1* transcript was detected using qRT-PCR from 1 to 15 days post infiltration (dpi). *N*. *benthamiana* plants agroinfiltrated with TYLCCNV/TYLCCNB displayed mild leaf curling symptoms at 3–5 dpi and the symptoms were severe from 6 dpi (Fig 1A). Compared with the control plants that were agroinfiltrated with the empty vector pBinPLUS (mock control), the expression of the *NbWRKY1* transcript in the systemic leaves of *N*. *benthamiana* inoculated with TYLCCNV/TYLCCNB was significantly higher than that of the control from 6 dpi (Fig 1B), suggesting that TYLCCNV/TYLCCNB infection upregulates the accumulation of the *NbWRKY1* mRNA in *N*. *benthamiana* plants.

### NbWRKY1 positively regulates plant defense against TYLCCNV/TYLCCNB

**Fig 2:**
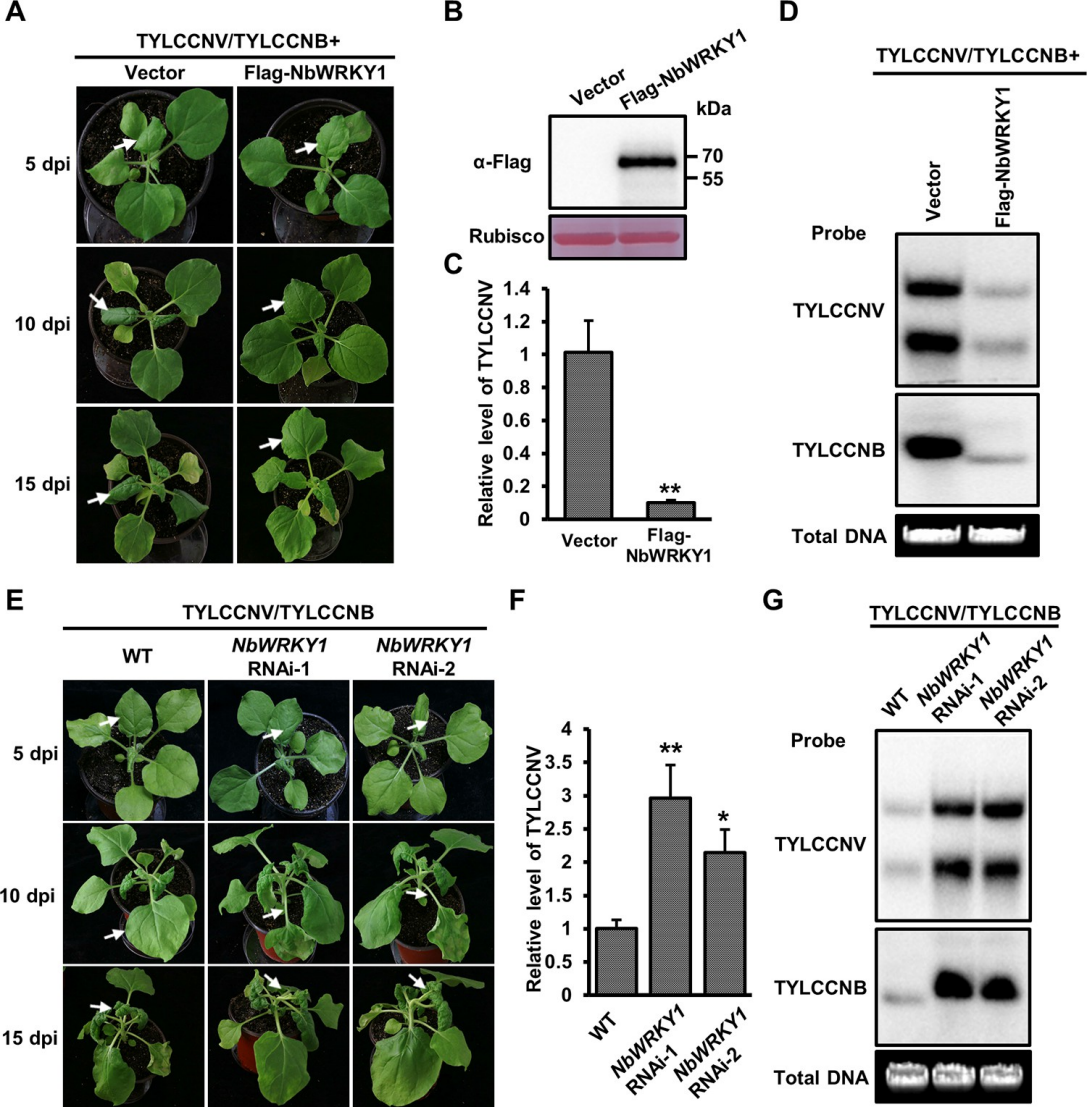
NbWRKY1 positively regulates plant defense against TYLCCNV/TYLCCNB. (A) Effect of the transient overexpression of NbWRKY1 on the symptoms induced by TYLCCNV/TYLCCNB. *N*. *benthamiana* plants were inoculated with TYLCCNV/TYLCCNB and Flag-NbWRKY1 or TYLCCNV/TYLCCNB and pCambia-Flag (the vector control) and symptoms were monitored and recorded at 5, 10 and 15 dpi, respectively. (B) Western blot analysis of the expression of Flag-NbWRKY1 in the infiltrated leaf samples at 48 hours post infiltration (hpi). (C) Quantitative PCR (qPCR) analysis of the relative accumulation of TYLCCNV DNA in the plants shown in (A) at 10 dpi. (D) Southern blot analysis of viral DNA accumulation in the systemic leaves of the inoculated plants as shown in (A) at 10 dpi. (E) Symptoms of the TYLCCNV/TYLCCNB-inoculated wild-type (WT) and *NbWRKY1* RNAi *N*. *benthamiana* plants at 5, 10, and 15 dpi, respectively. (F) qPCR analysis of the relative accumulation of TYLCCNV DNA in the plants shown in (E) at 10 dpi. (G) Southern blot analysis of viral DNA accumulation in the systemic leaves of the inoculated plants as shown in (E) at 10 dpi. White arrows indicate different severity of the corresponding leaves. 25S rRNA was used as an internal control of qPCR. Mean and standard deviation of four independent plants are shown. Double and single asterisks indicate significant statistical differences between two treatments at *p*<0.01 and *p*<0.05 based on Student’s *t* test, respectively. The Gelstain-stained agarose gel serves as a loading control.

To understand the function of NbWRKY1 in TYLCCNV/TYLCCNB infection, we generated a transient overexpression construct of NbWRKY1 (Flag-NbWRKY1) driven by the cauliflower mosaic virus (CaMV) 35S promoter. We then inoculated *N*. *benthamiana* plants with TYLCCNV/TYLCCNB and Flag-NbWRKY1 or with TYLCCNV/TYLCCNB and pCambia-Flag (the vector control) and monitored them over time to evaluate the effect of transient overexpression of NbWRKY1 on TYLCCNV/TYLCCNB infection. Transient overexpression of NbWRKY1 in *N*. *benthamiana* delayed the timing of symptom appearance and attenuated the symptom severity. At 5 dpi, the TYLCCNV/TYLCCNB and pCambia-Flag-inoculated *N*. *benthamiana* plants started to display mild leaf curling symptoms, whereas no obvious symptoms were observed in *N*. *benthamiana* plants infiltrated with TYLCCNV/TYLCCNB and Flag-NbWRKY1 (Fig 2A). Attenuation of symptom development by transient overexpression of Flag-NbWRKY1 was readily apparent at 10 dpi and 15 dpi (Fig 2A). Western blot analysis of the infiltrated leaf samples at 48 hours post infiltration (hpi) demonstrated the expression of Flag-NbWRKY1 (Fig 2B). Quantitative PCR (qPCR) and Southern blot analyses of viral DNA accumulation in the systemic leaves of the inoculated plants at 10 dpi showed that *N*. *benthamia*na plants infiltrated with TYLCCNV/TYLCCNB and Flag-NbWRKY1 accumulated less viral DNA when compared with the control plants (Fig 2C and 2D).

We also suppressed the expression of *NbWRKY1* using the tobacco rattle virus (TRV)-based virus-induced gene silencing (VIGS) system. As previously described [28], the NbWRKY1-silenced *N*. *benthamiana* plants (TRV-NbWRKY1) did not show clear growth defects compared with the non-silenced control plants (TRV-GFP) at 9 dpi. After confirming the silencing efficiency of NbWRKY1 in the NbWRKY1-silenced *N*. *benthamiana* plants (S1A Fig), we then inoculated the upper newly emerging leaves of these plants with the infectious clone of TYLCCNV/TYLCCNB. 10 days later, the TYLCCNV/TYLCCNB-inoculated TRV-NbWRKY1 *N*. *benthamiana* plants showed aggravated leaf curling symptoms as compared to the TRV-GFP plants inoculated with TYLCCNV/TYLCCNB (S1B Fig). Consistent with the severity of symptoms, viral DNA accumulation was significantly higher in the NbWRKY1-silenced plants than in the non-silenced plants (S1C Fig), suggesting that silencing of NbWRKY1 facilitates TYLCCNV/TYLCCNB infection.

We then generated two transgenic RNA interference (RNAi) lines for NbWRKY1 (referred to as *NbWRKY1* RNAi-1 and *NbWRKY1* RNAi-2, respectively). No difference in overall development was observed compared with the wild-type *N*. *benthamiana* plants (S2A Fig). qRT-PCR analysis of the *NbWRKY1* transcript showed that the expression of *NbWRKY1* was suppressed in the two transgenic RNAi lines (S2B Fig). After inoculation of these plants with the infectious clone of TYLCCNV/TYLCCNB through agroinfiltration, more severe leaf curling symptoms appeared on both *NbWRKY1* RNAi-1 and *NbWRKY1* RNAi-2 *N*. *benthamiana* plants than that appeared on the wild-type plants at either 5, 10, or 15 dpi (Fig 2E). qPCR and Southern blot analysis of viral DNA accumulation in the systemic leaves of the inoculated plants at 10 dpi showed that the accumulation of TYLCCNV/TYLCCNB in NbWRKY1 RNAi lines was markedly higher than that in wild-type *N*. *benthamiana* (Fig 2F and 2G). These results suggested that NbWRKY1 is a positive regulator of plant defense against TYLCCNV/TYLCCNB.

### NbWRKY1 binds to and inactivates the NbWHIRLY1 promoter

**Fig 3:**
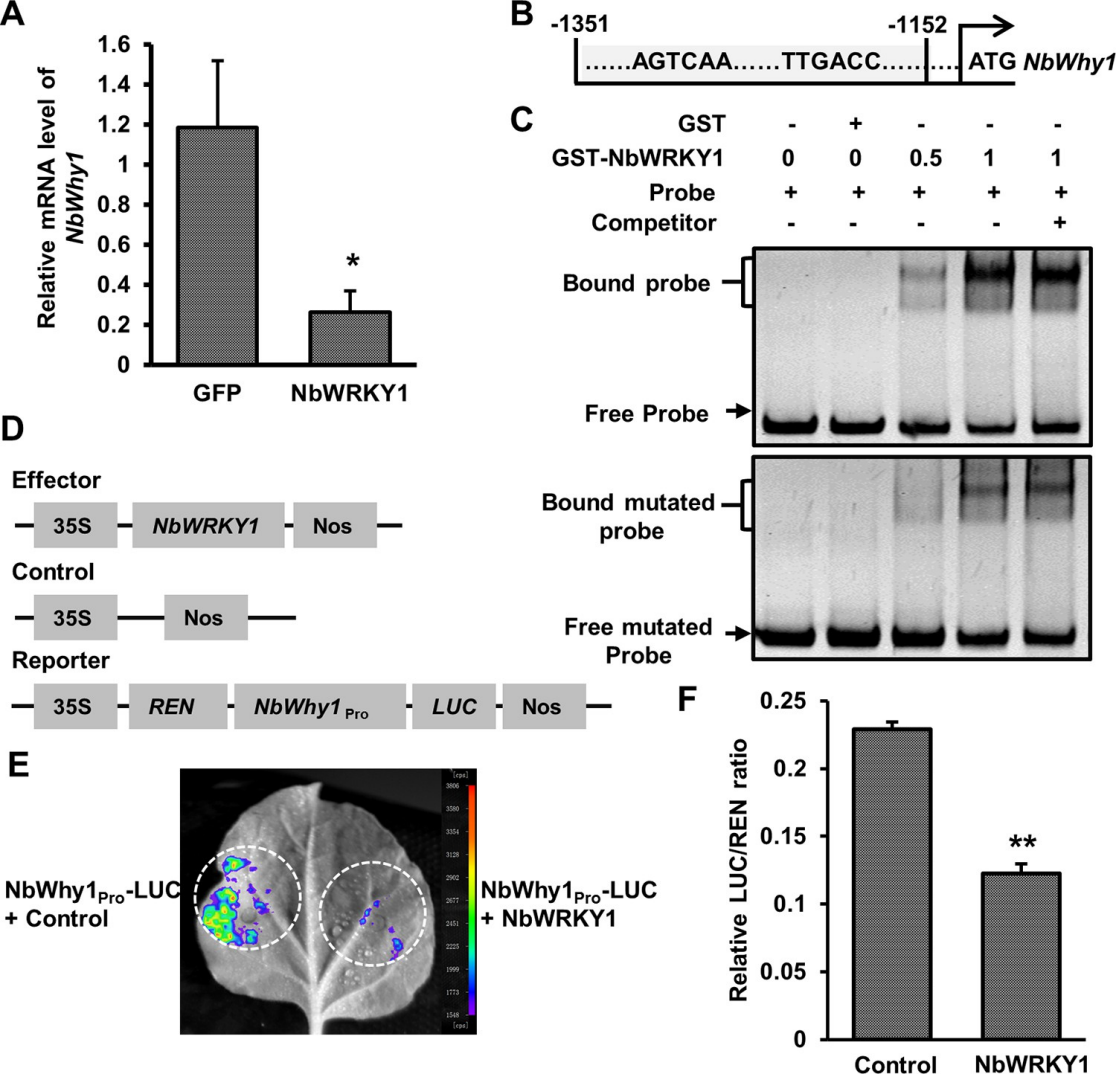
NbWRKY1 binds to the NbWHIRLY1 (NbWhy1) promoter and represses NbWhy1 transcription. **(**A) RT-qPCR analysis of *NbWhy1* mRNA level in *N*. *benthamiana* plants transiently overexpressing NbWRKY1. *NbGAPDH* was used as an internal control. Mean and standard deviation of three independent plants are shown. Asterisk indicates a significant statistical difference between two treatments at *p*<0.05 based on Student’s *t* test. (B) Schematic depiction of the putative W-box elements found in the promoter region of *NbWhy1*. The region from -1351 to -1152 nucleotides upstream of the ATG codon of *NbWRKY1* was used for electrophoretic mobility shift assay (EMSA). (C) EMSA showing the specific binding of NbWRKY1 to the *NbWhy1* promoter. Purified GST or GST-NbWRKY1 protein from *Escherichia coli* extracts was incubated with 20 ng of DNA probes. Poly (dI-dC) was used in excess amounts (100-fold) as non-specific competitor. The lower panel indicates the mutation of W-box in the probe. (D) Schematic depiction of the transient luciferase reporter and effector constructs. (E) NbWRKY1-mediated repression of transcription. Expression of luciferase (LUC) reporter was driven by the *NbWhy1* promoter (*NbWhy1*_Pro_). The luciferase fluorescence in the infiltrated *N*. *benthamiana* leaves was detected by the live molecular imaging system. 35S, the 35S promoter of cauliflower mosaic virus; REN, Renilla luciferase; Nos, Nos terminator. (F) Measurement of relative LUC/REN ratio in *N*. *benthamiana* leaves. Mean and standard deviation of three independent replicates are shown. Double asterisks indicates a significant statistical difference between two treatments at *p*<0.01 based on Student’s *t* test.

Considering that WRKYs are plant-specific transcription factors, we wondered whether NbWRKY1 interacts with other plant-specific transcription factors to control the expression of target genes. We reviewed the literatures about these types of transcription factors, such as AP2/ERF, NAC, and WHIRLY. As RNA silencing is an important antiviral defense in plants, we were particularly interested in WHIRLY1, a single-stranded DNA-binding protein that has been described to regulate microRNA levels during stress [29]. We cloned and obtained the full-length coding sequence of NbWhy1 from *N*. *benthamiana* plants. Sequence analysis showed that NbWhy1 belongs to the ES I group of WHIRLY and contains a transcriptional activation region and chloroplast transport peptide in the N-terminus, a single-stranded DNA binding domain and nuclear localization signal in the WHIRLY domain, and the C-terminal domain, respectively (S3 Fig). qRT-PCR experiments showed that the expression of *NbWhy1* was downregulated in *N*. *benthamiana* plants transiently overexpressing NbWRKY1 (Fig 3A).

We also cloned and analyzed the promoter of NbWhy1. The promoter of the NbWhy1 contains a potential NbWRKY1 binding element or W-box sequences (TTGACC/T) that implies a direct interaction between NbWRKY1 and the promoter of NbWhy1 (Fig 3B). Therefore, we conducted an electrophoretic mobility shift assay (EMSA) using 200 bp of the NbWhy1 promoter region (-1351 to -1152 bp upstream of the NbWhy1 start codon) with either purified GST or recombinant GST-NbWRKY1 protein. A retarded band was observed when purified GST-NbWRKY1 protein but not the GST protein was mixed with a DNA probe that contained the W-box sequences (Fig 3C). The retarded band was also observed when the nonspecific competitor poly (dI-dC) was used to minimize nonspecific interactions, indicating the specific binding of NbWRKY1 to the NbWhy1 promoter (Fig 3C). Mutagenesis of the W-box of NbWhy1 promoter attenuates the binding of NbWRKY1 to NbWhy1 (Fig 3C), suggesting that NbWRKY1 might regulate NbWhy1 expression by binding to the W-box motif.

Next, we utilized a dual-luciferase reporter (DLR) assay to investigate the impact of NbWRKY1 on NbWhy1 expression. We co-expressed a luciferase (LUC) reporter driven by the NbWhy1 promoter (*NbWhy1Pro*-LUC) with NbWRKY1 or the control vector pGreenII 62 SK in *N*. *benthamiana* leaves. The resultant LUC activity indicated that the NbWhy1 promoter effectively drove the expression of the reporter gene, which in turn was repressed by the expression of NbWRKY1 (Fig 3D and 3E). Compared to the pGreenII 62 SK control, the LUC-to-Renilla luciferase (REN) (LUC/REN) ratio was decreased by approximately 50% in the presence of NbWRKY1 (Fig 3F). Collectively, these data suggest that NbWRKY1 binds to the NbWhy1 promoter and inactivates the promoter activity of NbWhy1.

### NbWhy1 negatively regulates plant resistance against TYLCCNV/TYLCCNB

**Fig 4:**
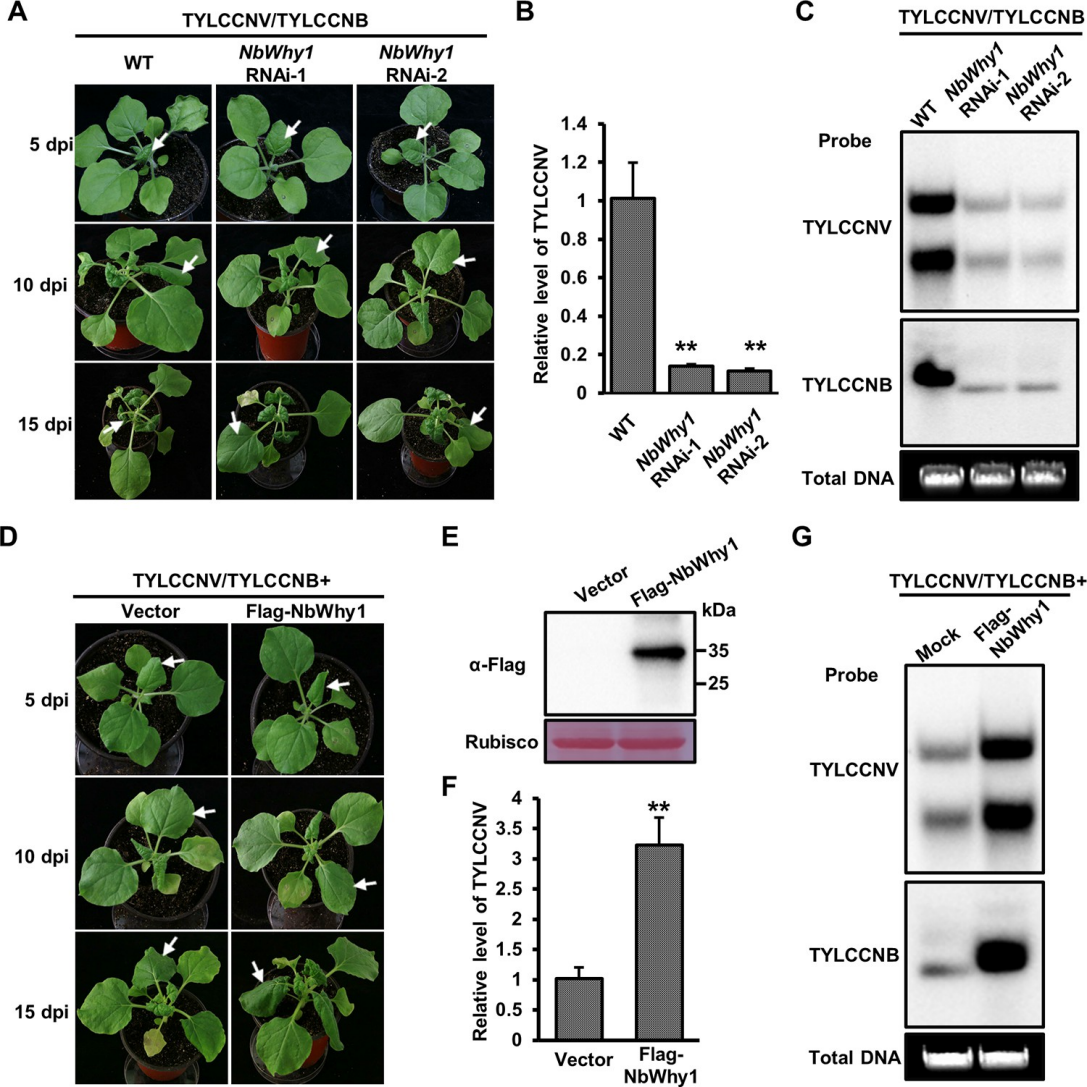
NbWhy1 negatively regulates plant defense against TYLCCNV/TYLCCNB. (A) Symptoms of the TYLCCNV/TYLCCNB-inoculated wild-type (WT) and *NbWhy1* RNAi *N*. *benthamiana* plants at 5, 10, and 15 dpi, respectively. (B) qPCR analysis of the relative accumulation of TYLCCNV DNA in the plants shown in (A) at 10 dpi. (C) Southern blot analysis of viral DNA accumulation in the systemic leaves of the inoculated plants as shown in (A) at 10 dpi. The Gelstain-stained agarose gel serves as a loading control. (D) Effect of the transient overexpression of NbWhy1 on the symptoms induced by TYLCCNV/TYLCCNB. *N*. *benthamiana* plants were inoculated with TYLCCNV/TYLCCNB and Flag-NbWhy1 or TYLCCNV/TYLCCNB and pCambia-Flag (the vector control) and symptoms were monitored and recorded at 5, 10 and 15 dpi, respectively. (E) Western blot analysis of the expression of Flag-NbWhy1 in the infiltrated leaf samples at 48 hours post infiltration (hpi). (F) qPCR analysis of the relative accumulation of TYLCCNV DNA in the plants shown in (D) at 10 dpi. (G) Southern blot analysis of viral DNA accumulation in the systemic leaves of the inoculated plants as shown in (D) at 10 dpi. White arrows indicate different severity of the corresponding leaves. 25S rRNA was used as an internal control of qPCR. Mean and standard deviation of four independent plants are shown. Double asterisks indicates a significant statistical difference between two treatments at *p*<0.01 based on Student’s *t* test. The Gelstain-stained agarose gel serves as a loading control.

Since NbWRKY1 binds to the NbWhy1 promoter and downregulates NbWhy1 transcription, we determined whether NbWhy1 plays an opposite role in plant defense against TYLCCNV/TYLCCNB. We generated two transgenic RNA interference (RNAi) lines for NbWhy1 (referred to as *NbWhy1* RNAi-1 and *NbWhy1* RNAi-2, respectively). The NbWhy1-silenced *N*. *benthamiana* plants did not show clear growth defects compared with the wildtype *N*. *benthamiana* plants (S4 Fig). We then inoculated these RNAi plants with the infectious clone of TYLCCNV/TYLCCNB through agroinfiltration; wild-type *N*. *benthamiana* plants (WT) inoculated with TYLCCNV/TYLCCNB were used as controls. Milder leaf curling symptoms appeared on both *NbWhy1* RNAi-1 and *NbWhy1* RNAi-2 plants compared to those in the WT plants at either 5, 10, or 15 dpi (Fig 4A). qPCR and southern blot analysis of viral DNA accumulation in the systemic leaves of the inoculated plants at 10 dpi showed that the accumulation of TYLCCNV/TYLCCNB in *NbWhy1* RNAi lines was significantly less than that in wild-type *N*. *benthamiana* (Fig 4B and 4C). Meanwhile, we constructed a transient overexpression construct of NbWhy1 (Flag-NbWhy1) under the 35S promoter and inoculated *N*. *benthamiana* plants with TYLCCNV/TYLCCNB plus Flag-NbWhy1 or TYLCCNV/TYLCCNB plus pCambia-Flag (vector control) to evaluate the effect of transient overexpression of NbWhy1 on TYLCCNV/TYLCCNB infection. We found that transient overexpression of NbWhy1 in *N*. *benthamiana* accelerated symptom severity as compared to the plants inoculated with TYLCCNV/TYLCCNB and pCambia-Flag (Fig 4D and 4E). *N*. *benthamia*na plants infiltrated with TYLCCNV/TYLCCNB and Flag-NbWhy1 accumulated more viral DNA when compared with the control plants inoculated with TYLCCNV/TYLCCNB and pCambia-Flag (Fig 4F and 4G). Therefore, our data suggested that NbWhy1 negatively regulates plant defense against TYLCCNV/TYLCCNB.

### NbWhy1 impairs RNAi and interferes with NbCaM3-NbCAMTA3 interaction

**Fig 5:**
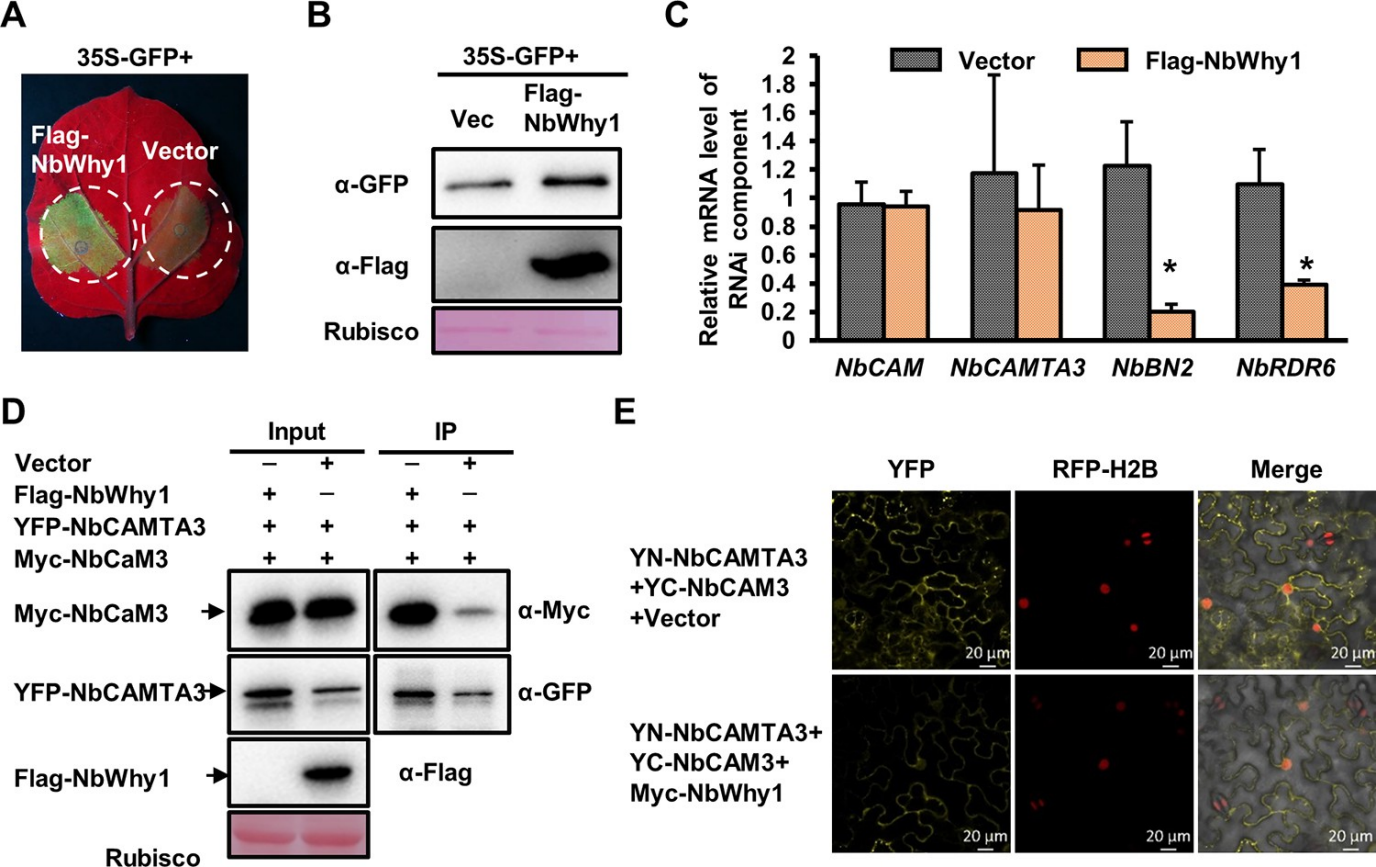
NbWhy1 impairs RNAi and interferes with NbCaM3-NbCAMTA3 interaction. (A) GFP fluorescence in the leaf patches of *N*. *benthamiana* infiltrated with 35S-GFP+Flag-NbWhy1 or 35S-GFP+pCambia-Flag. Infiltrated leaves were photographed under UV light at 3 dpi. (B) Western blot analysis of GFP accumulation and expression of Nbwhy1 in the infiltrated patches of (A) using the anti-GFP and anti-Flag antibodies, respectively. Rubisco staining was used as the loading control. (C) qRT-PCR analysis of the mRNA level of key RNAi components in *N*. *benthamiana* leaves infiltrated with Flag-NbWhy1 or the pCambia-Flag vector at 48 hpi. *NbGAPDH* was used as an internal control. Mean and standard deviation of three independent plants are shown. Asterisk indicates a significant statistical difference between two treatments at *p*<0.05 based on Student’s *t* test. (D) Co-immunoprecipitation assays showing NbWhy1-mediated attenuation of the interaction between NbCaM3 and NbCAMTA3. *N*. *benthamiana* leaves were infiltrated with Agrobacterium cells harboring the constructs, as indicated. Blots with input samples (Input) or immunoprecipitated samples (IP) were detected with anti-GFP, or anti-Flag, or anti-Myc antibodies. Arrows show the expected protein bands. (E) BiFC assays showing the impaired interaction between NbCAM3 and NbCAMTA3 by NbWhy1. Images were captured using a Zeiss LSM 880 confocal laser scanning microscope at 48 hpi. RFP-H2B served as a nuclear marker. Bars represent 20 μm.

As RNA silencing is an important antiviral defense and WHIRLY1 regulates microRNA levels during stress [29], we wondered whether NbWhy1 regulates RNAi in plants. We co-expressed Flag-NbWhy1 or pCambia-Flag with GFP in *N*. *benthamiana* plants. At 3 days post infiltration, GFP fluorescence was stronger in leaves overexpressing Flag-NbWhyl than those expressing pCambia-Flag (Fig 5A). Western blot analysis showed that the GFP protein level was higher in plants overexpressing Flag-NbWhy1 than in control plants (Fig 5B), suggesting that NbWhy1 negatively regulates RNAi in plants.

To understand how NbWhy1 targets RNAi, we transiently expressed Flag-NbWhy1 in *N*. *benthamiana* leaves via agrobacterium-mediated infiltration and detected the expression of key components of the RNA silencing pathway at 48 hpi using qRT-PCR. We found that transient overexpression of NbWhy1 does not affect the expression of *NbCaM3* and *NbCAMTA3* transcripts, but it significantly reduced the expression of the *NbBN2* and *NbRDR6* transcripts (Fig 5C). As a previous study demonstrated that NbCaM3-NbCAMTA3 interaction activated CAMTA3-mediated transcriptional activation of both RDR6 and BN2 [27], we wondered whether NbWhy1 impacts the NbCaM3-NbCAMTA3 interaction. To this end, we infiltrated *N*. *benthamiana* leaves with *Agrobacterium* containing Flag-NbWhy1, Myc-NbCaM3, and YFP-NbCAMTA3 or pCambia-Flag, Myc-NbCaM3, and YFP-NbCAMTA3, and detected the interaction between NbCaM3 and NbCAMTA3 at 48 hpi. As previously described, NbCaM3 interacted with NbCAMTA3 [27]. However, less Myc-NbCaM3 was immunoprecipitated by YFP-NbCAMTA3 in the presence of Flag-NbWhy1 (Fig 5D). BiFC assays showed that Myc-tagged NbWhy1, but not the Myc control, impaired the NbCaM3-NbCAMTA3 interaction (Fig 5E). These results indicated that NbWhy1 interferes with the interaction of NbCaM3 and NbCAMTA3 to impair RNAi in plants.

### NbWRKY1 enhances plant defense against TYLCCNV/TYLCCCNB by repressing NbWhy1

**Fig 6:**
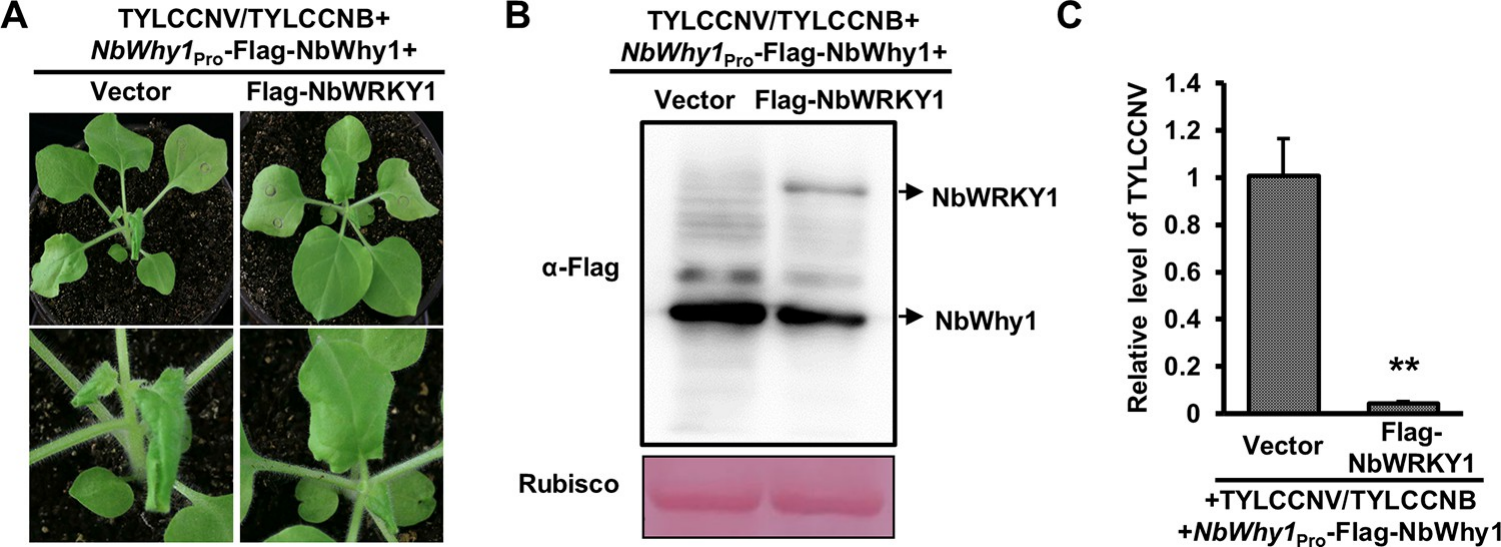
NbWRKY1 enhances plant defense against TYLCCNV/TYLCCCNB by repressing NbWhy1. (A) Symptoms induced by TYLCCNV/TYLCCNB in *N*. *benthamiana* plants inoculated with TYLCCNV/TYLCCNB+*NbWhy1*_Pro_-Flag-NbWhy1+Flag-NbWRKY1 or TYLCCNV/TYLCCNB+*NbWhy1*_Pro_-Flag-NbWhy1+pCambia-Flag (Vector) at 5 dpi. (B) Western blot analysis of the expression of Flag-NbWhy1 and Flag-NbWRKY1 in the infiltrated leaf samples at 48 hpi. Ponceau staining of Rubisco serves as a loading control. (C) qPCR analysis of the relative accumulation of TYLCCNV DNA in the plants shown in (A). 25S rRNA was used as an internal control. Mean and standard deviation of three independent plants are shown. Double asterisks indicates a significant statistical difference between two treatments at *p*<0.01 based on Student’s *t* test.

Based on the knowledge of NbWRKY1 inhibits the promoter activity of NbWhy1 and NbWRKY1 and NbWhy1 have opposite roles in regulating plant defense against TYLCCNV/TYLCCNB, we proposed that NbWRKY1 binding to the NbWhy1 promoter repressed the function of NbWhy1 during TYLCCNV/TYLCCNB infection. We infiltrated *N*. *benthamiana* plants with TYLCCNV/TYLCCNB+NbWhy1pro-NbWhy1+Flag-NbWRKY1 or TYLCCNV/TYLCCNB+NbWhy1pro-NbWhy1+pCambia-Flag (control). We found that *N*. *benthamiana* plants displayed much weaker symptoms and accumulated significantly less viral DNA when compared to the control plants infiltrated with TYLCCNV/TYLCCNB+NbWhy1pro-NbWhy1+pCambia-Flag (Fig 6), suggesting that co-expression of NbWRKY1 with NbWhy1 significantly suppressed TYLCCNV/TYLCCNB infection.

### NbWRKY1-NbWhy1 cassette confers plant defense against TYLCV

**Fig 7:**
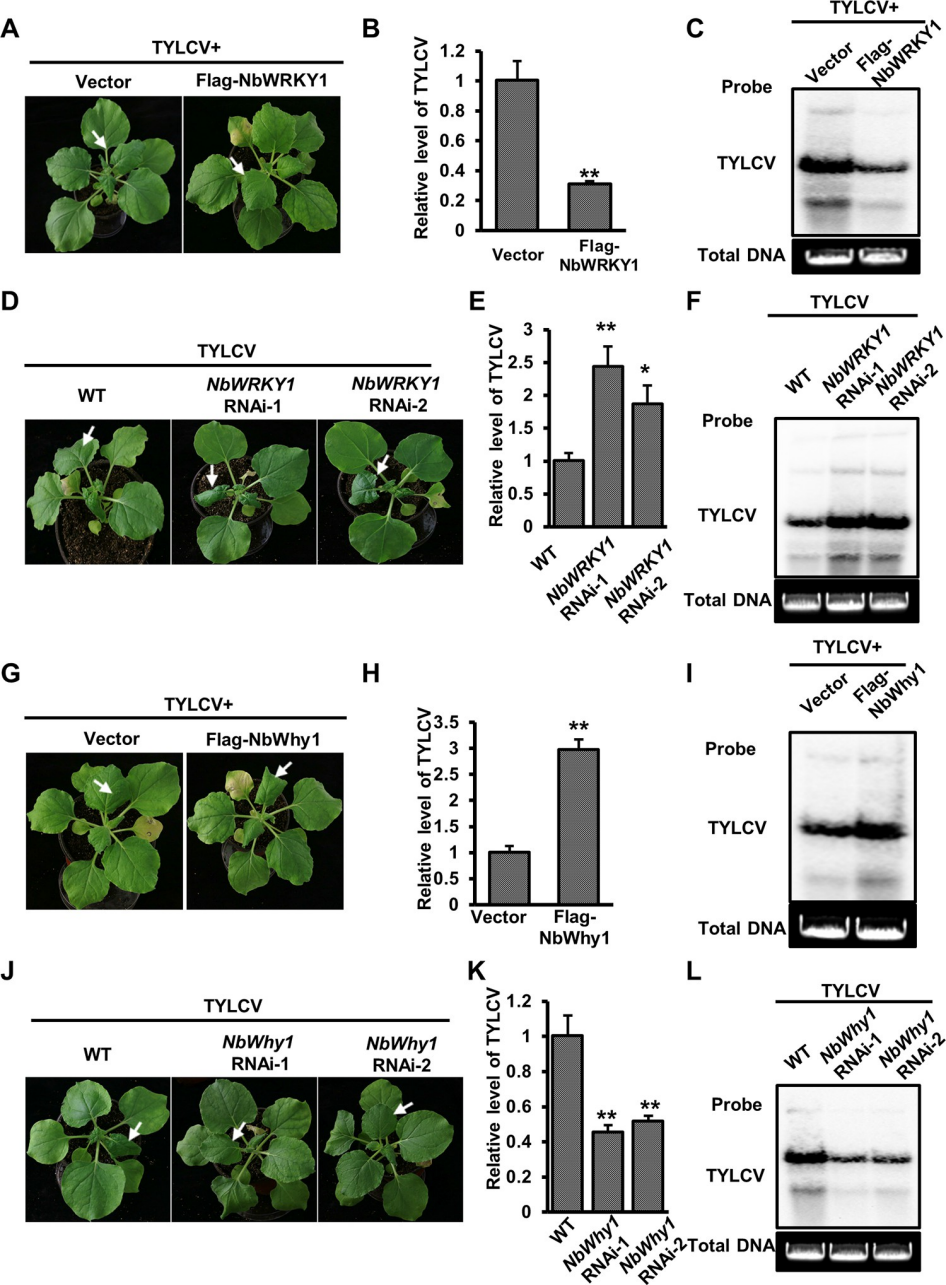
The NbWRKY1-NbWhy1 cassette combats tomato yellow leaf curl virus (TYLCV) infection. **. (A)** Effect of the transient overexpression of NbWRKY1 on the symptoms induced by TYLCV at 14 dpi. *N*. *benthamiana* plants inoculated with TYLCV and pCambia-Flag (the vector control) were used as controls. (B) qPCR analysis of the relative accumulation of TYLCV DNA in the plants shown in (A). (C) Southern blot analysis of viral DNA accumulation in the systemic leaves of the inoculated plants as shown in (A). (D) Symptoms of TYLCV-inoculated wild-type (WT) and *NbWRKY1* RNAi *N*. *benthamiana* plants at 14 dpi. (E) qPCR analysis of the relative accumulation of TYLCV DNA in the plants shown in (D). (F) Southern blot analysis of viral DNA accumulation in the systemic leaves of the inoculated plants as shown in (D). (G) Effect of the transient overexpression of NbWhy1 on the symptoms induced by TYLCV at 14 dpi. (H) qPCR analysis of the relative accumulation of TYLCV DNA in the plants shown in (G). (I) Southern blot analysis of viral DNA accumulation in the systemic leaves of the inoculated plants as shown in (G). (J) Symptoms of TYLCV-inoculated wild-type (WT) and *NbWhy1* RNAi *N*. *benthamiana* plants at 14 dpi. (K) qPCR analysis of the relative accumulation of TYLCV DNA in the plants shown in (J). (L) Southern blot analysis of viral DNA accumulation in the systemic leaves of the inoculated plants as shown in (J). White arrows indicate different severity of the corresponding leaves. 25S rRNA was used as an internal control of qPCR. Mean and standard deviation of four independent plants are shown. Double and single asterisks indicate significant statistical differences between two treatments at *p*<0.01 and *p*<0.05 based on Student’s *t* test, respectively. The Gelstain-stained agarose gel serves as a loading control.

To explore the relevance of the NbWRKY1-NbWhyl cassette in other geminiviruses, we chose TYLCV, a globally prevalent monopartite geminivirus, to determine the role of NbWRKY1 and NbWhy1 in TYLCV infection. Firstly, we used qRT-PCR to detect the expression of *NbWRKY1* following TYLCV infection. We found that the transcript level of *NbWRKY1* in the systemic leaves of *N*. *benthamiana* inoculated with TYLCV was significantly higher than that of the control from 8 dpi (S5 Fig). We then inoculated *N*. *benthamiana* plants with the infectious clone of TYLCV with Flag-NbWRKY1 or pCambia-Flag (control) by agroinfiltration. Compared to plants that were inoculated with TYLCV and pCambia-Flag, the plants inoculated with TYLCV and Flag-NbWRKY1 displayed milder leaf curling symptoms and accumulated less viral DNA (Figs 7A–7C and S6A). We also inoculated *NbWRKY1* RNAi and wild-type *N*. *benthamiana* plants with TYLCV. TYLCV caused more severe leaf curling symptoms in *NbWRKY1* RNAi plants than in wild-type plants, accompanied by more viral DNA as indicated by qPCR and gel blot, respectively (Figs 7D–7F and S6B). These results indicate that NbWRKY1 is a positive regulator of plant defense against TYLCV.

We also inoculated *N*. *benthamiana* plants with the infectious clone of TYLCV with Flag-NbWhy1 or pCambia-Flag. A much more severe leaf curling symptoms and higher viral DNA accumulation were observed in *N*. *benthamiana* plants inoculated with TYLCV and Flag-NbWhy1 when compared to the plants that were inoculated with TYLCV and pCambia-Flag (Figs 7G–7I and S6C). Conversely, the *NbWhy1* RNAi lines dramatically attenuated the symptoms induced by TYLCV and reduced viral DNA accumulation when compared with the wild-type *N*. *benthamiana* plants, as indicated by qPCR and gel blot (Figs 7J–7L and S6D). These results indicate NbWhy1 is a susceptible factor of TYLCV. As NbWRKY1 inhibits the transcription of NbWhy1, our results indicate that the NbWRKY1-NbWhy1 module confers plant defense against TYLCV infection.

## Discussion

Plants are equipped with a sophisticated set of antiviral defenses to limit geminivirus infection. Transcription factors are key masters of signaling transduction that enable the rapid mounting of plant defense response. Our recent work has demonstrated that NbWRKY1 is involved in incompatible plant-geminivirus interaction and silencing of NbWRKY1 attenuates the cell death triggered by MMDaV or the core RepA protein encoded by MMDaV in *N*. *benthamiana*. We have also found that transient expression of RepA confers plant resistance to TYLCCNV/TYLCCNB and TYLCV infection [28]. This led us to hypothesize that the resistance against MMDaV could be potentially used by plants to improve resistance against different geminiviruses. Here, we demonstrate the positive regulatory role of NbWRKY1 in compatible plant-geminivirus interaction and describe a novel transcriptional cascade of NbWRKY1 involved in geminivirus infection.

The role of NbWRKY1 in compatible plant-geminivirus interaction was explored first. As was reported for incompatible plant-geminivirus interaction [28], expression of NbWRKY1 was induced in response to infection of TYLCCNV/TYLCCNB and TYLCV, two distinct begomoviruses of the family *Geminiviridae*, indicating that upregulation of NbWRKY1 might be a general phenomenon during geminivirus infection. We further showed that transient overexpression of NbWRKY1 results in enhanced plant defense against TYLCCNV/TYLCCNB and TYLCV whereas RNAi of NbWRKY1 enhances plant susceptibility to these two geminiviruses, suggesting that NbWRKY1 is a positive regulator of plant defense against virus infection.

We further identified NbWhy1 as a potential downstream component of NbWRKY1. As shown by EMSA, NbWRKY1 binds to the promoter sequences of NbWhy1. The NbWhy1 promoter contains one W-box motif, mutation of the W-box of NbWhy1 promoter attenuates but not abolishes the binding of NbWRKY1 to NbWhy1, suggesting that the W-box might not be the only area of NbWhy1 promoter where NbWRKY1 binding occurs (Fig 3). A promoter transient assay indicates that NbWRKY1 inactivates NbWhy1 expression, which was also demonstrated by the reduced NbWhy1 transcripts upon transient overexpression of NbWRKY1 in *N*. *benthamiana* plants. In agreement with this, RNAi of *NbWhy1* dramatically attenuated geminivirus infection and transient overexpression of NbWhy1 increased plant susceptibility to geminivirus, supporting that NbWhy1 negatively regulates plant responses to geminiviruses. The finding that the function of NbWhy1 in enhancing geminivirus infection was suppressed by the overexpression of NbWRKY1 further demonstrated that NbWRKY1 represses NbWhy1.

WHIRLIES are plant-specific proteins that can bind to single-stranded DNA in plastids, mitochondria, and nucleus. They are divided into five groups based on phylogenetic analysis and whether they have transcriptional activation regions or not [30]. WHIRLIES have been identified to be involved in plant resistance to diverse abiotic and biotic stresses. Previous report showed that several plant species overexpressing WHIRLIES have a higher resistance toward stress and pathogen attacks. For example, StWhy1, a member of the ES I group of the WHIRLY family, activates the expression of PR-10a and induces immune responses in potato [31]. Likewise, AtWhy1 plays a positive regulatory role in basal and induced gene defense responses in Arabidopsis [32,33]. Nevertheless, the role of WHIRLY transcription factor in plant virus infection was not investigated. The NbWhy1 identified here belongs to the ES I group of the WHIRLY family. Our study demonstrated that NbWhy1 is a negative regulator of plant response to geminiviruses, which extends the functionality of WHIRLY to plant antiviral response. On the other hand, we identified that WHIRLY act as a negative regulator of plant response toward a pathogen attack, which could be engineered to attenuate geminivirus infection.

This study also reveals that NbWhy1 negatively regulates the RNA silencing pathway, as indicated by the GFP leaf patch assays. Expression profile of several RNAi genes indicated that NbWhy1 repressed the transcript of *NbRDR6* and *NbBN2*. Further analysis proved that NbWhy1 disrupted the interaction between NbCaM3 and NbCAMTA3 to inhibit the expression of downstream genes such as NbRDR6 and NbBN2, though how NbWhy1 interferes with the CaM3-CAMTA3 interaction remains to be understood. Although a previous study showed that WHIRLY1 regulates the level of stress-responsive microRNAs (miRNA) in barley, it remains to be explored whether WHIRLY affects the biosynthesis and/or stability of miRNAs by directly binding to nuclear miRNAs and/or through its impact on other plant factors [29]. NbBN2 is a bifunctional ribonuclease that degrades AGO1/AGO2 and DCL1-targeting miRNAs, which leads to upregulation of *AGO1*, *AGO2*, and *DCL* mRNA levels [33–37]. The finding that NbWhy1 represses the expression of *NbBN2* implies that NbWhy1 might affect the miRNA stability in *N*. *benthamiana* plants.

**Fig 8:**
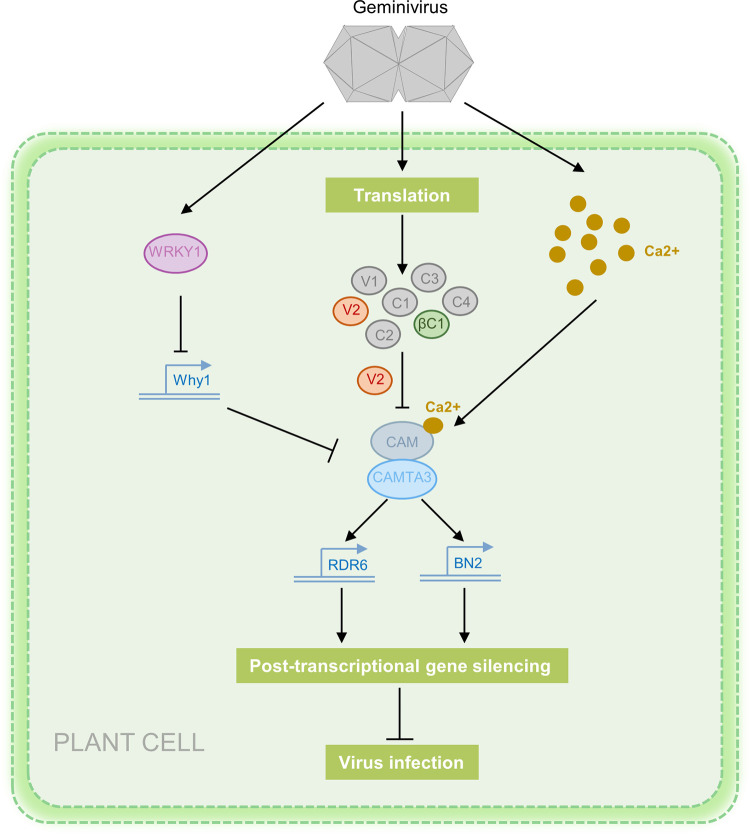
A working model summarizing the role of NbWRKY1 in regulation of geminivirus infection. Geminivirus infection could activate the Ca2+ pool and trigger Ca2+→CaMs→CAMTA3→BN2/RDR6 signaling cascade to prime antiviral RNAi defense. To counteract host defense, geminivirus-encoded V2 disrupts the CaM-CaMTA3 interaction to suppress RNA silencing [26]. To counter-counter defense, NbWRKY1 is upregulated upon geminivirus infection. NbWRKY1 represses the expression of NbWhy1, a negative regulator of RNAi that can interfere with the CaM3-CAMTA3, leading to enhanced plant defense against geminivirus infection. Arrows represents activation while the “T” sign indicates attenuation or suppression.

Given that *NbWRKY1* is upregulated in response to geminivirus infection and NbWRKY1 suppresses the transcription of *NbWhy1*, we initially raised a possibility that geminivirus infection downregulates the transcript level of *NbWhy1*. However, RT-qPCR analysis of the transcript level of *NbWhy1* during TYLCCNV/TYLCCNB or TYLCV infection showed that TYLCCNV/TYLCCNB and TYLCV infection did not significantly alter the expression of the *NbWhy1* transcript at early-infection stages. On the contrary, their infection increased the transcript level of *NbWhy1* at late-infection stages (S7 Fig). As geminiviruses employ different strategies to counteract plant antiviral responses during the co-evolutionary arms race, we speculate that geminiviruses might interfere with the NbWRKY1-NbWhy1 module to establish a successful infection. Previously the βC1 protein encoded by TYLCCNB was reported to disrupt the dimerization of AtWRKY20 and the interaction between AtWRKY20-ORA59 to suppress WRKY20 activity and thus benefitting whitefly but deterring nonvector insect herbivores [14]. It is remained to be answered whether geminiviral proteins, such as βC1 and V2, interfere with the NbWRKY1 activity or disrupt the binding of NbWRKY1 to the NbWhy1 promoter. *N*. *benthamiana* is a universal susceptible host for many plant viruses due to, at least in part, the presence of a loss-of-function mutation in an RNA-dependent RNA polymerase gene (*NbRDR1m*) of its genome [38]. It would be interesting to investigate whether the WRKY1-Why1 module-mediated antiviral response is conversed in other plant-geminivirus interactions. Based on our results, we propose a working model for NbWRKY1-mediated plant defense against geminivirus infection (Fig 8). Geminivirus infection stimulates the expression of *NbWRKY1*. NbWRKY1 binds to the NbWhy1 promoter and represses the expression of *NbWhy1*, a negative regulator of RNAi that disrupts the interaction between NbCAM and NbCAMTA3 to promote geminivirus infection. These findings pave the way to a deeper understanding of the combinational interaction between transcription factor and the antiviral RNA defense and complicated defense and counter-defense interplays during geminivirus infection, which could be further employed to control geminiviruses.

## Materials and methods

### Plant materials

Wild type, RFP-H2B transgenic, *NbWRKY1*-silenced, and *NbWhy1*-silenced *Nicotiana benthamiana* plants were cultivated and grown in an insect-free greenhouse at 25°C with a photoperiod of 16 h light/8 h darkness. Generally, 4-week-old *N*. *benthamiana* plants were used for assays.

### Plasmid construction

To generate the construct used for transient expression of NbWRKY1, NbWRKY1 was amplified and inserted between *BamH*I and *Sal*I sites of a modified pCambia1307 vector containing the 3×Flag coding sequence generate pCambia-3×Flag-NbWRKY1 (referred to Flag-NbWRKY1 thereafter) as described [39]. To obtain the recombinant plasmid pCambia-3×Flag-NbWhy1 (Flag-NbWhy1) used for transient expression of NbWhy1, the full-length NbWhy1 fragment was amplified and seamlessly cloned to the pCambia-3×Flag vector using the ClonExpress II one step cloning kit (Vazyme, Beijing, China). Recombinant plasmids 201-YN-NbCAMTA3 (YN-NbCAMTA3), 201-YC-NbCAM3 (YC-NbCAM3), pBA-Flag-Myc4-NbWhy1 (Myc-NbWhy1), pEarleyGate 103-NbWRKY1 (NbWRKY1-GFP), pBA-Flag-Myc4-NbCaM3 (Myc-NbCaM3), and pEarleyGate 104-NbCAMTA3 (YFP-NbCAMTA3), which were used for transient expression, were constructed using the standard protocols of the Gateway system as per the manufacturer’s instructions (Invitrogen, Carlsbad, CA, USA). To generate RNAi constructs for NbWRKY1 and NbWhy1, partial-length sequence of NbWRKY1 and NbWhy1 were amplified using Flag-NbWRKY1 and Flag-NbWhy1 as templates, respectively. The sense and complementary sense fragments of NbWRKY1 or NbWhy1 were cloned into the pRNAi-LIC-L vector using seamless cloning as instructed (Vazyme). Recombinant plasmids were transformed into *Agrobacterium tumefaciens* EHA105 by electroporation. Primers used in the construction of the above recombinant constructs are listed in S1 Table.

### Agrobacterium-mediated virus inoculation and transient gene expression

*A*. *tumefaciens* harboring the infectious clone of TYLCCNV/TYLCCNB or TYLCV [40,41] were cultured, pelleted, and diluted to OD_600_ = 1.0 using the infiltration buffer (10 mM MgCl_2_, 10 mM MES, pH5.6, and 100 μM acetosyringone). Fully expanded leaves of *N*. *benthamiana* plants, as indicated in figure legends, were infiltrated with the infectious clone of TYLCCNV/TYLCCNB or TYLCV using a needless syringe as described [42]. The infiltrated plants were observed at various days post inoculation and were photographed with a Canon 530D digital camera.

For virus-induced gene silencing (VIGS) assay, TRV-based VIGS system was used as described [43]. About 300-bp DNA fragment of NbWhy1 was PCR amplified and cloned into the TRV2 vector to obtain TRV2-NbWhy1. *A*. *tumerfaciens* culture carrying TRV1 or the derivatives of TRV2 (TRV2-GFP, TRV2-NbWRKY1 [28], and TRV2-NbWhy1) were diluted to OD_600_ = 0.5. Equal volumes of the diluted *A*. *tumerfaciens* cultures carrying TRV1 were mixed with those carrying TRV2-GFP, or TRV2-NbWRKY1, or TRV2-NbWhy1 prior to infiltration.

Transient gene expression and leaf patch assays were conducted in *N*. *benthamiana* leaves via agroinfiltration as described previously [44].

### RNA extraction and quantitative reverse transcription PCR (qRT-PCR)

Total RNA was extracted from systemically infected *N*. *benthamiana* plants with TRIzol reagent (Invitrogen) and reverse transcribed into cDNA using the PrimeScript RT Reagent Kit with gDNA Eraser (TaKaRa, Dalian, China) as described [45]. The diluted cDNA was used as template and the qRT-PCR was conducted using the RealStar Green Fast Mixture (Genstar, Beijing, China) and the LightCycler 96 system (Roche Diagnostics, Rotkreuz, Switzerland). The glyceraldehyde 3-phosphate dehydrogenase (GAPDH) was used as the internal controls for qPCR and qRT-PCR assays, respectively. Relative expression of target genes was evaluated using the comparative *Ct* method (2^-ΔΔCt^) [46]. Primers used in the qRT-PCR assays were listed in S1 Table.

### Total DNA extraction, quantitative polymerase chain reaction, and Southern blotting

Total plant DNA was isolated from infected young leaves of *N*. *benthamiana* plants using the CTAB method as described previously [42]. For quantitative analysis of viral DNA accumulation, quantitative polymerase chain reaction (qPCR) was performed essentially as the above mentioned qRT-PCR, except that DNA was used as template and viral DNA accumulation was normalized to the expression of 25S rRNA. For Southern blotting, the RNAase-treated DNA was separated by 1% agarose gel electrophoresis and stained with ethidium bromide to show equal sample loadings. After denaturation and neutralization, total DNA was transferred to Hybond N^+^ nylon membranes (GE Healthcare, Pittsburgh, PA, USA) and cross-linked with UV cross linker. Partial fragments of viral DNA were labelled with digoxigenin using the DIG High Prime DNA Labeling and Detection Starter Kit (Roche). Hybridization was performed at 55°C and the signal of DNA blotting was determined using a chemiluminescence detection system (Tianneng, Shanghai, China).

### Protein extraction and Western blot assay

Total protein was extracted from infiltrated leaf patches of *N*. *benthamiana* as described. Western blot analysis was performed as described using anti-GFP monoclonal antibody (Roche), or anti-Myc polyclonal antibody (Genscript, Piscataway, NJ, USA), or anti-βC1 monoclonal antibody (made in our laboratory), or anti-Flag M2 monoclonal antibody (Sigma, Los Angeles, USA). A secondary horseradish peroxidase-conjugated goat anti-mouse antibody (EASYBIO, Beijing, China) was used. The chemiluminescence chromogenic solution (Tanon High-sig ECL Western Blotting Substrate) and a chemiluminescence detection system (Tianneng) were used for visualization of the signal of blotted proteins.

### Dual-luciferase reporter assay

Dual-luciferase reporter assay was carried out in *N*. *benthamiana* plant leaves as described [47]. The ~1750-bp promoter sequence of NbWhy1 was amplified from genomic DNA of *N*. *benthamiana* plant leaves and seamlessly cloned into the pGreenII 0800-LUC vector predigested with *BamH*I and *Sal*I to generate the reporter plasmid 35S::REN-NbWhy1Pro::LUC. The full-length fragment of NbWRKY1 was amplified and inserted to pGreenII 62-SK through *BamH*I and *Sal*I to yield the effector plasmid 35S:NbWRKY1. The effector and reporter plasmids were transformed into *Agrobacterium* strain GV3101 containing the pSoup plasmid. *A*. *tumefaciens* cells harboring the effector and reporter plasmid were equally mixed and infiltrated into 4-week-old *N*. *benthamiana* leaves. At 48 h post infiltration, the underside of the inoculated *N*. *benthamiana* leaves was sprayed with 1 mM luciferin substrate D-Luciferin Potassium Salt reaction solution (Biovision, CA, USA) and the images were captured using live molecular imaging system. LUC activities were measured using the Dual-Luciferase Reporter Assay System (Promega) with a GLOMAX96 microplate luminometer (Promega) as described previously [48].

### Electrophoretic mobility shift assay

Electrophoretic mobility shift assay (EMSA) was performed as reported previously [49]. The recombinant proteins GST and GST-NbWRKY1 were expressed in *Escherichia coli* (BL21), purified with ProteinIso GST Resin (TransGen, Beijing, China), and eluted with 10 mM reduced glutathione (GSH) following the manufacturer’s instructions (TransGen). DNA fragments were synthesized and incubated with approximately 100 μg of purified GST, or GST-NbWRKY1 protein in a 20 μL binding reaction system (20 mM Tris base, 2 mM dithiothreitol, 5 mM MgCl_2_, 0.5 μg calf BSA, and 5% (v/v) glycerol) at room temperature for 15 min. For the competition assays, 1 μg of nonspecific competitor poly(dI-dC) was added to the reaction. The reaction mixtures were electrophoresed on a 5% non-denaturing polyacrylamide gel electrophoresis for 1–2 hours at room temperature.

### Bimolecular fluorescence complementation, and co-immunoprecipitation assays

Bimolecular fluorescence complementation (BiFC) assays were conducted as described [42]. Images of fluorescent proteins in the epidermal cells of agroinfiltrated RFP-H2B plants were collected using a laser confocal microscope (LSM880; Carl Zeiss, Jena, Germany) at 48 h post infiltration. YFP was excited at 488 nm and emission was captured at 497–520 nm. RFP was excited at 561 nm and emission was captured at 585–615 nm. For each experimental sample, at least three independent biological replicates were examined. Images were processed with ZEN software (Zeiss).

Co-immunoprecipitation assays were performed on protein exacts from *N*. *benthamiana* leaves collected at 2 dpi by using GFP-Trap_MA (Chromotek, Hauppauge, NY, USA) as previously described [50]. Anti-Myc polyclonal antibody (Genscript, Piscataway, NJ, USA) was used at a 1:3000 dilution for immunoblot analysis. Blotted membranes were washed thoroughly and visualized using a chemiluminescence detection system (Tianneng).

The numerical data used in all figures are included in S1 Data.

## Supporting information

S1 Data Excel spreadsheet containing, in separate sheets, the underlying numerical data and statistical analysis for Figure panels 1B, 2C, 2F, 3A, 3F, 4B, 4F, 5C, 6C, 7B, 7E, 7H, 7K, S1A, S1C, S2B, S4B, S5, S7A, and S7B. (XLSX)

S1 Table Primers used in this study. (XLSX)

S1 Fig Tobacco rattle virus (TRV)-induced silencing of NbWRKY1 promotes TYLCCNV/TYLCCNB infection in *Nicotiana benthamiana* plants. (A) qRT-PCR analysis of the silencing efficiency of NbWRKY1 in TRV-GFP and TRV-NbWRKY1 inoculated *N. benthamiana* plants. *NbGAPDH* was used as an internal control. Mean and standard deviation of three independent plants are shown. (B) Effect of NbWRKY1 silencing on the symptoms induced by TYLCCNV/TYLCCNB. *N. benthamiana* plants were first infiltrated with TRV1 and TRV2 derivate as indicated. *N. benthamiana* plants infiltrated with TRV1 and TRV2-GFP were used as a control. After 10 days, the upper leaves were infiltrated with TYLCCNV/TYLCCNB. Photos were taken at 10 dpi. (C) qPCR analysis of the relative accumulation of TYLCCNV DNA in the plants shown in (B) at 10 dpi. 25S rRNA was used as an internal control. Mean and standard deviation of four independent plants are shown. Double asterisks indicates a significant statistical difference between two treatments at *p*<0.01 based on Student’s t test.
(TIF)

S2 Fig Phenotype and silencing efficiency of transgenic NbWRKY1 RNAi plants. (A) The growth of 5-week-old wild-type (WT) and *NbWRKY1* RNAi *N*. *benthamiana* plants. (B) qRT-PCR analysis of the silencing efficiency of NbWRKY1 in plants used in (A). *NbGAPDH* was used as an internal control. Mean and standard deviation of four independent plants are shown. Double asterisks indicates a significant statistical difference between two treatments at *p*<0.01 based on Student’s t test.
(TIF)

S3 Fig Sequence analysis of NbWhy1. (A) Schematic representation of the structure of NbWhy1. The predicted conserved domains were shown as indicated. TAR, transcriptional activation region; CTP, chloroplast transport peptide; ssDNA, single-stranded DNA binding domain; NLS, nuclear localization signal. (B) Phylogenetic tree representing relationships of *Nicotiana benthamiana* NbWhy1 to WHIRLY transcription factors from different plant species. The phylogenetic tree was constructed based on amino acid sequences of WHIRLIES using the neighbor-joining method in MEGA7.0. Accession numbers for each WRKY transcription factor are indicated.
(TIF)

S4 Fig Phenotype and silencing efficiency of transgenic NbWhy1 RNAi plants. (A) The growth of 5-week-old wild-type (WT) and *NbWhy1* RNAi *N*. *benthamiana* plants. (B) qRT-PCR analysis of the silencing efficiency of NbWhy1 in plants used in (A). *NbGAPDH* was used as an internal control. Mean and standard deviation are shown. Double asterisks indicates a significant statistical difference between two treatments at *p*<0.01 based on Student’s t test.
(TIF)

S5 Fig qRT-PCR analysis of the expression level of *NbWRKY1* in plants inoculated with the infectious clone of TYLCV or mock-inoculated plants. RNA was extracted from the upper non-inoculated plant leaves at various days post inoculation (dpi) as indicated. *NbGAPDH* was used as an internal control. Asterisks indicate significant statistical differences between two treatments at *p*<0.05 based on Student’s *t* test.
(TIF)

S6 Fig Effect of NbWRKY1 and NbWhy1 on symptoms induced by TYLCV. (A) Effect of transient overexpression of NbWRKY1 on the symptoms induced by TYLCV. *N*. *benthamiana* plants were inoculated with TYLCV and Flag-NbWRKY1 or TYLCV and pCambia-Flag (the vector control) and symptoms were monitored and recorded at 7 and 21 dpi, respectively. (B) Symptoms of the TYLCV-inoculated wild-type (WT) and NbWRKY1 RNAi *N*. *benthamiana* plants at 7 and 21 dpi, respectively. (C) Effect of transient overexpression of NbWhy1 on the symptoms induced by TYLCV. *N*. *benthamiana* plants were inoculated with TYLCV and Flag-NbWhy1 or TYLCV and pCambia-Flag (the vector control) and symptoms were monitored and recorded at 7 and 21 dpi, respectively. (D) Symptoms of the TYLCV-inoculated wild-type (WT) and NbWhy1 RNAi *N*. *benthamiana* plants at 7 and 21 dpi, respectively. White arrows indicate different severity of the corresponding leaves.
(TIF)

S7 Fig qRT-PCR analysis of the expression level of *NbWhy1* in response to TYLCCNV/TYLCCNB or TYLCV infection. *N*. *benthamiana* plants were inoculated with the infectious clone of TYLCCNV/TYLCCNB or TYLCV. Plants inoculated with the empty vector were used as mock controls. RNA was extracted from the upper non-inoculated plant leaves at various days post inoculation (dpi) as indicated. *NbGAPDH* was used as an internal control. Mean and standard deviation of three independent plants are shown. Double and single asterisks indicate significant statistical differences between two treatments at *p*<0.01 and *p*<0.05 based on Student’s *t* test, respectively.
(TIF)

## References

[1] Liu Y, Yang T, Lin Z, Gu B, Xing C, Zhao L, et al. A WRKY transcription factor PbrWRKY53 from *Pyrus betulaefolia* is involved in drought tolerance and AsA accumulation. Plant Biotechnol J. 2019;17(9):1770–87. Epub 2019/02/26. doi: 10.1111/pbi.13099 30801865 PMC6686137

[2] Shrestha A, Khan A, Dey N. cis-trans engineering: advances and perspectives on customized transcriptional regulation in plants. Mol Plant. 2018;11(7):886–98. Epub 2018/06/03. doi: 10.1016/j.molp.2018.05.008 29859265

[3] Rushton PJ, Somssich IE, Ringler P, Shen QJ. WRKY transcription factors. Trends Plant Sci. 2010;15(5):247–58. doi: 10.1016/j.tplants.2010.02.006 20304701

[4] Eulgem T, Rushton PJ, Robatzek S, Somssich IE. The WRKY superfamily of plant transcription factors. Trends Plant Sci. 2000;5(5):199–206. Epub 2000/04/29. doi: 10.1016/s1360-1385(00)01600-9 10785665

[5] Rushton PJ, Bokowiec MT, Han S, Zhang H, Brannock JF, Chen X, et al. Tobacco transcription factors: novel insights into transcriptional regulation in the Solanaceae. Plant Physiol. 2008;147(1):280–95. Epub 2008/03/14. doi: 10.1104/pp.107.114041 18337489 PMC2330323

[6] Zhang Y, Wang L. The WRKY transcription factor superfamily: its origin in eukaryotes and expansion in plants. BMC Evol Biol. 2005;5:1. Epub 2005/01/05. doi: 10.1186/1471-2148-5-1 15629062 PMC544883

[7] Jiang J, Ma S, Ye N, Jiang M, Cao J, Zhang J. WRKY transcription factors in plant responses to stresses. J Integr Plant Biol. 2017;59(2):86–101. doi: 10.1111/jipb.12513 27995748

[8] Wani SH, Anand S, Singh B, Bohra A, Joshi R. WRKY transcription factors and plant defense responses: latest discoveries and future prospects. Plant Cell Rep. 2021;40(7):1071–85. doi: 10.1007/s00299-021-02691-8 33860345

[9] Phukan UJ, Jeena GS, Shukla RK. WRKY Transcription Factors: Molecular regulation and stress responses in plants. Front Plant Sci. 2016;7:760. Epub 2016/07/05. doi: 10.3389/fpls.2016.00760 27375634 PMC4891567

[10] Hwang SH, Kwon SI, Jang JY, Fang IL, Lee H, Choi C, et al. OsWRKY51, a rice transcription factor, functions as a positive regulator in defense response against *Xanthomonas oryzae* pv. oryzae. Plant Cell Rep. 2016;35(9):1975–85. Epub 2016/06/15. doi: 10.1007/s00299-016-2012-0 27300023

[11] Choi N, Im JH, Lee E, Lee J, Choi C, Park SR, et al. WRKY10 transcriptional regulatory cascades in rice are involved in basal defense and *Xa1*-mediated resistance. J Exp Bot. 2020;71(12):3735–48. Epub 2020/04/01. doi: 10.1093/jxb/eraa135 32227093

[12] Zheng Z, Qamar SA, Chen Z, Mengiste T. *Arabidopsis* WRKY33 transcription factor is required for resistance to necrotrophic fungal pathogens. Plant J. 2006;48(4):592–605. Epub 2006/10/25. doi: 10.1111/j.1365-313X.2006.02901.x . 17059405

[13] Chen L, Zhang L, Li D, Wang F, Yu D. WRKY8 transcription factor functions in the TMV-cg defense response by mediating both abscisic acid and ethylene signaling in *Arabidopsis*. Proc Natl Acad Sci U S A. 2013;110(21):E1963–71. Epub 2013/05/08. doi: 10.1073/pnas.1221347110 23650359 PMC3666684

[14] Zhao P, Yao X, Cai C, Li R, Du J, Sun Y, et al. Viruses mobilize plant immunity to deter nonvector insect herbivores. Sci Adv. 2019;5:eaav9801. Epub 2019/08/21. doi: 10.1126/sciadv.aav9801 31457079 PMC6703867

[15] Cai M, Qiu D, Yuan T, Ding X, Li H, Duan L, et al. Identification of novel pathogen-responsive cis-elements and their binding proteins in the promoter of OsWRKY13, a gene regulating rice disease resistance. Plant Cell Environ. 2008;31(1):86–96. Epub 2007/11/08. doi: 10.1111/j.1365-3040.2007.01739.x 17986178

[16] Chi Y, Yang Y, Zhou Y, Zhou J, Fan B, Yu JQ, et al. Protein-protein interactions in the regulation of WRKY transcription factors. Mol Plant. 2013;6(2):287–300. Epub 2013/03/05. doi: 10.1093/mp/sst026 23455420

[17] Rojas MR, Macedo MA, Maliano MR, Soto-Aguilar M, Souza JO, Briddon RW, et al. World management of geminiviruses. Annu Rev Phytopathol. 2018;56:637–77. Epub 2018/08/29. doi: 10.1146/annurev-phyto-080615-100327 30149794

[18] Fiallo-Olivé E, Lett JM, Martin DP, Roumagnac P, Varsani A, Zerbini FM, et al. ICTV virus taxonomy profile: *Geminiviridae* 2021. J Gen Virol. 2021;102(12). Epub 2021/12/18. doi: 10.1099/jgv.0.001696 34919512 PMC8744271

[19] Li F, Qiao R, Wang Z, Yang X, Zhou X. Occurrence and distribution of geminiviruses in China. Sci China Life Sci. 2022;65:1498–503. Epub 2022/06/02. doi: 10.1007/s11427-022-2125-2 35661965

[20] Aguilar E, Garnelo Gomez B, Lozano-Duran R. Recent advances on the plant manipulation by geminiviruses. Curr Opin Plant Biol. 2020;56:56–64. Epub 2020/05/29. doi: 10.1016/j.pbi.2020.03.009 32464465

[21] Li F, Yang X, Bisaro DM, Zhou X. The βC1 protein of geminivirus-betasatellite complexes: a target and repressor of host defenses. Mol Plant. 2018;11(12):1424–6. Epub 2018/11/08. doi: 10.1016/j.molp.2018.10.007 30404041

[22] Yang X, Guo W, Li F, Sunter G, Zhou X. Geminivirus-associated betasatellites: exploiting chinks in the antiviral arsenal of plants. Trends Plant Sci. 2019;24(6):519–29. Epub 2019/04/21. doi: 10.1016/j.tplants.2019.03.010 31003895

[23] Lopez-Gomollon S, Baulcombe DC. Roles of RNA silencing in viral and non-viral plant immunity and in the crosstalk between disease resistance systems. Nat Rev Mol Cell Biol. 2022;23(10):645–62. Epub 2022/06/18. doi: 10.1038/s41580-022-00496-5 35710830

[24] Baulcombe DC. The role of viruses in identifying and analyzing RNA silencing. Annu Rev Virol. 2022;9(1):353–73. Epub 2022/06/03. doi: 10.1146/annurev-virology-091919-064218 35655339

[25] Baulcombe D. RNA silencing in plants. Nature. 2004;431(7006):356–63. doi: 10.1038/nature02874 15372043

[26] Ding S-W. RNA-based antiviral immunity. Nat Rev Immunol 2010;10(9):632–44. doi: 10.1038/nri2824 20706278

[27] Wang Y, Gong Q, Wu Y, Huang F, Ismayil A, Zhang D, et al. A calmodulin-binding transcription factor links calcium signaling to antiviral RNAi defense in plants. Cell Host Microbe. 2021;29(9):1393–406.e7. Epub 2021/08/06. doi: 10.1016/j.chom.2021.07.003 34352216

[28] Sun S, Ren Y, Wang D, Farooq T, He Z, Zhang C, et al. A group I WRKY transcription factor regulates mulberry mosaic dwarf-associated virus-triggered cell death in *Nicotiana benthamiana*. Mol Plant Pathol. 2022;23(2):237–53. Epub 2021/11/06. doi: 10.1111/mpp.13156 34738705 PMC8743015

[29] Świda-Barteczka A, Krieger-Liszkay A, Bilger W, Voigt U, Hensel G, Szweykowska-Kulinska Z, et al. The plastid-nucleus located DNA/RNA binding protein WHIRLY1 regulates microRNA-levels during stress in barley (*Hordeum vulgare* L.). RNA Biol. 2018;15(7):886–91. Epub 2018/06/28. doi: 10.1080/15476286.2018.1481695 29947287 PMC6161701

[30] Desveaux D, Maréchal A, Brisson N. Whirly transcription factors: defense gene regulation and beyond. Trends Plant Sci. 2005;10(2):95–102. Epub 2005/02/15. doi: 10.1016/j.tplants.2004.12.008 15708347

[31] Desveaux D, Després C, Joyeux A, Subramaniam R, Brisson N. PBF-2 is a novel single-stranded DNA binding factor implicated in PR-10a gene activation in potato. Plant Cell. 2000;12(8):1477–89. Epub 2000/08/19. doi: 10.1105/tpc.12.8.1477 10948264 PMC149117

[32] Desveaux D, Subramaniam R, Després C, Mess JN, Lévesque C, Fobert PR, et al. A "Whirly" transcription factor is required for salicylic acid-dependent disease resistance in *Arabidopsis*. Dev Cell. 2004;6(2):229–40. Epub 2004/02/13. doi: 10.1016/s1534-5807(04)00028-0 14960277

[33] Tao Y, Xie Z, Chen W, Glazebrook J, Chang HS, Han B, et al. Quantitative nature of Arabidopsis responses during compatible and incompatible interactions with the bacterial pathogen *Pseudomonas syringae*. Plant Cell. 2003;15(2):317–30. Epub 2003/02/05. doi: 10.1105/tpc.007591 12566575 PMC141204

[34] Allen E, Xie Z, Gustafson AM, Carrington JC. microRNA-directed phasing during trans-acting siRNA biogenesis in plants. Cell. 2005;121(2):207–21. Epub 2005/04/27. doi: 10.1016/j.cell.2005.04.004 15851028

[35] Pertermann R, Tamilarasan S, Gursinsky T, Gambino G, Schuck J, Weinholdt C, et al. A viral suppressor modulates the plant immune response early in infection by regulating microRNA activity. mBio. 2018;9(2):e00419–18. Epub 2018/04/25. doi: 10.1128/mBio.00419-18 29691336 PMC5915741

[36] Vaucheret H, Vazquez F, Crété P, Bartel DP. The action of ARGONAUTE1 in the miRNA pathway and its regulation by the miRNA pathway are crucial for plant development. Genes Dev. 2004;18(10):1187–97. Epub 2004/05/08. doi: 10.1101/gad.1201404 15131082 PMC415643

[37] Wu J, Yang Z, Wang Y, Zheng L, Ye R, Ji Y, et al. Viral-inducible Argonaute18 confers broad-spectrum virus resistance in rice by sequestering a host microRNA. Elife. 2015;4. Epub 2015/02/18. doi: 10.7554/eLife.05733 25688565 PMC4358150

[38] Yang SJ, Carter SA, Cole AB, Cheng NH, Nelson RS. A natural variant of a host RNA-dependent RNA polymerase is associated with increased susceptibility to viruses by *Nicotiana benthamiana*. Proc Natl Acad Sci U S A. 2004;101: 6297–6302. Epub 2004/04/12. doi: 10.1073/pnas.0304346101 15079073 PMC395963

[39] Cui Y, Fang X, Qi Y. TRANSPORTIN1 promotes the association of microRNA with ARGONAUTE1 in *Arabidopsis*. Plant Cell. 2016;28(10):2576–85. Epub 2016/09/23. doi: 10.1105/tpc.16.00384 27662897 PMC5134980

[40] Zhou X, Xie Y, Tao X, Zhang Z, Li Z, Fauquet CM. Characterization of DNAß associated with begomoviruses in China and evidence for co-evolution with their cognate viral DNA-A. J Gen Virol. 2003;84(Pt 1):237–47. Epub 2003/01/21. doi: 10.1099/vir.0.18608–0 12533720

[41] Zhang H, Gong H, Zhou X. Molecular characterization and pathogenicity of tomato yellow leaf curl virus in China. Virus Genes. 2009;39(2):249–55. Epub 2009/07/11. doi: 10.1007/s11262-009-0384-8 19590945

[42] Zhou T, Zhang M, Gong P, Li F, Zhou X. Selective autophagic receptor NbNBR1 prevents NbRFP1-mediated UPS-dependent degradation of ßC1 to promote geminivirus infection. PLoS Pathog. 2021;17(9):e1009956. Epub 2021/09/28. doi: 10.1371/journal.ppat.1009956 34570833 PMC8496818

[43] Liu Y, Schiff M, Dinesh-Kumar SP. Virus-induced gene silencing in tomato. Plant J. 2002;31(6):777–86. Epub 2002/09/11. doi: 10.1046/j.1365-313x.2002.01394.x 12220268

[44] Yang X, Guo W, Ma X, An Q, Zhou X. Molecular characterization of tomato leaf curl China virus, infecting tomato plants in China, and functional analyses of its associated betasatellite. Appl Environ Microbiol. 2011;77(9):3092–101. Epub 2011/03/08. doi: 10.1128/AEM.00017-11 21378048 PMC3126389

[45] Li F, Zhao N, Li Z, Xu X, Wang Y, Yang X, et al. A calmodulin-like protein suppresses RNA silencing and promotes geminivirus infection by degrading SGS3 via the autophagy pathway in *Nicotiana benthamiana*. PLoS Pathog. 2017;13(2):e1006213. Epub 2017/02/18. doi: 10.1371/journal.ppat.1006213 28212430 PMC5333915

[46] Livak KJ, Schmittgen TD. Analysis of relative gene expression data using real-time quantitative PCR and the 2(-Delta Delta C(T)) Method. Methods. 2001;25(4):402–8. Epub 2002/02/16. doi: 10.1006/meth.2001.1262 11846609

[47] Zhang F, Fang H, Wang M, He F, Tao H, Wang R, et al. APIP5 functions as a transcription factor and an RNA-binding protein to modulate cell death and immunity in rice. Nucleic Acids Res. 2022;50(9):5064–79. Epub 2022/05/08. doi: 10.1093/nar/gkac316 35524572 PMC9122607

[48] Hellens RP, Allan AC, Friel EN, Bolitho K, Grafton K, Templeton MD, et al. Transient expression vectors for functional genomics, quantification of promoter activity and RNA silencing in plants. Plant Methods. 2005;1:13. Epub 2005/12/18. doi: 10.1186/1746-4811-1-13 16359558 PMC1334188

[49] Li C, He H, Wang J, Liu H, Wang H, Zhu Y, et al. Characterization of a LAL-type regulator NemR in nemadectin biosynthesis and its application for increasing nemadectin production in *Streptomyces cyaneogriseus*. Sci China Life Sci. 2019;62(3):394–405. Epub 2019/01/29. doi: 10.1007/s11427-018-9442-9 30689104

[50] Li F, Zhang C, Li Y, Wu G, Hou X, Zhou X, et al. Beclin1 restricts RNA virus infection in plants through suppression and degradation of the viral polymerase. Nat Commun. 2018;9(1):1268. Epub 2018/03/30. doi: 10.1038/s41467-018-03658-2 29593293 PMC5871769

