# Supplementary figures and images for: WRKY1 represses the WHIRLY1 transcription factor to positively regulate plant defense against geminivirus infection
Source: PLoS Pathog. 2023 Apr 7;19(4):e1011319. doi: 10.1371/journal.ppat.1011319 (PMC10115308; doi:10.1371/journal.ppat.1011319)

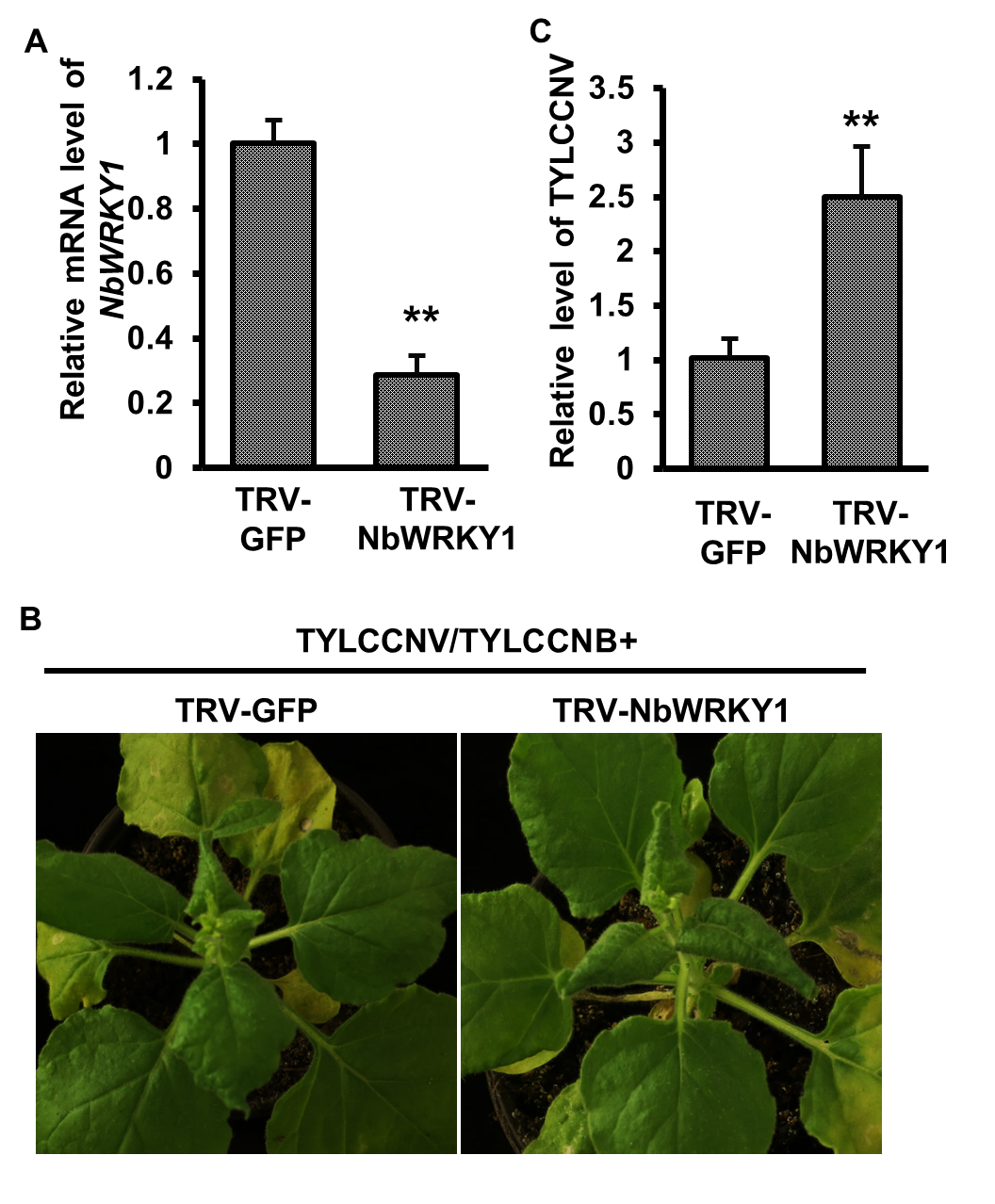

Supplement: S1 Fig — (A) qRT-PCR analysis of the silencing efficiency of NbWRKY1 in TRV-GFP and TRV-NbWRKY1 inoculated N. benthamiana plants. NbGAPDH was used as an internal control. Mean and standard deviation of three independent plants are shown. (B) Effect of NbWRKY1 silencing on the symptoms induced by TYLCCNV/TYLCCNB. N. benthamiana plants were first infiltrated with TRV1 and TRV2 derivate as indicated. N. benthamiana plants infiltrated with TRV1 and TRV2-GFP were used as a control. After 10 days, the upper leaves were infiltrated with TYLCCNV/TYLCCNB. Photos were taken at 10 dpi. (C) qPCR analysis of the relative accumulation of TYLCCNV DNA in the plants shown in (B) at 10 dpi. 25S rRNA was used as an internal control. Mean and standard deviation of four independent plants are shown. Double asterisks indicates a significant statistical difference between two treatments at p<0.01 based on Student’s t test. (TIF) [file ppat.1011319.s003.tif]

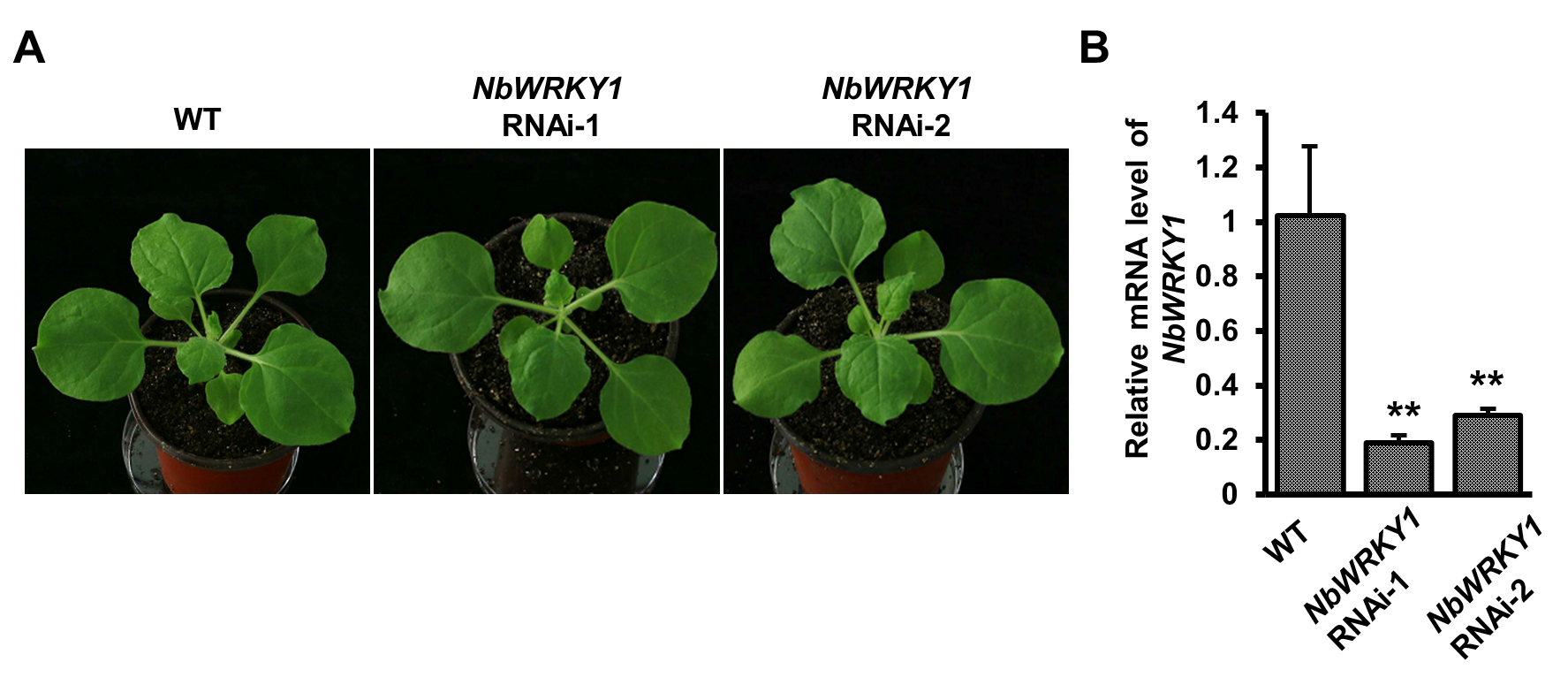

Supplement: S2 Fig — (A) The growth of 5-week-old wild-type (WT) and NbWRKY1 RNAi N. benthamiana plants. (B) qRT-PCR analysis of the silencing efficiency of NbWRKY1 in plants used in (A). NbGAPDH was used as an internal control. Mean and standard deviation of four independent plants are shown. Double asterisks indicates a significant statistical difference between two treatments at p<0.01 based on Student’s t test. (TIF) [file ppat.1011319.s004.tif]

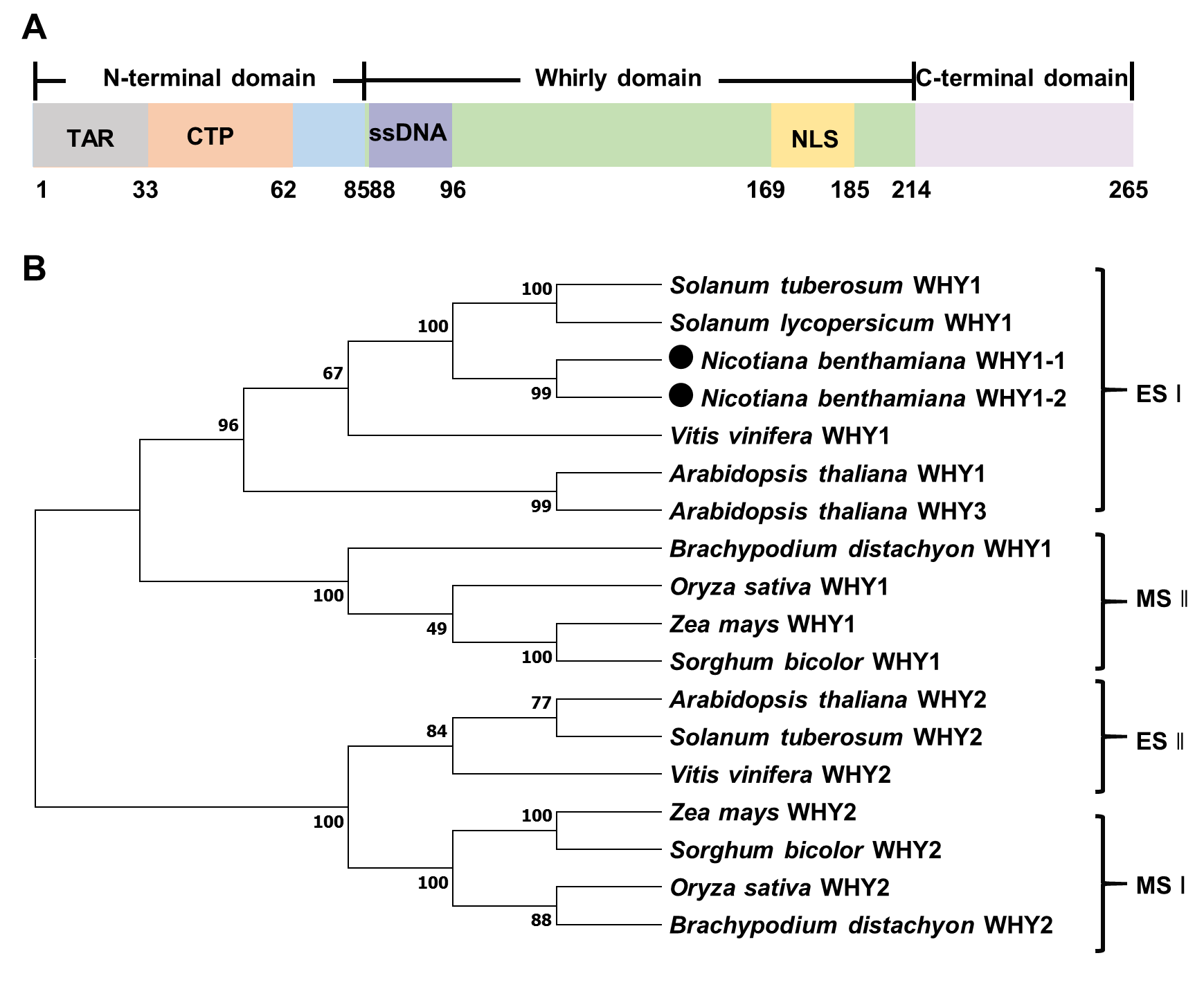

Supplement: S3 Fig — (A) Schematic representation of the structure of NbWhy1. The predicted conserved domains were shown as indicated. TAR, transcriptional activation region; CTP, chloroplast transport peptide; ssDNA, single-stranded DNA binding domain; NLS, nuclear localization signal. (B) Phylogenetic tree representing relationships of Nicotiana benthamiana NbWhy1 to WHIRLY transcription factors from different plant species. The phylogenetic tree was constructed based on amino acid sequences of WHIRLIES using the neighbor-joining method in MEGA7.0. Accession numbers for each WRKY transcription factor are indicated. (TIF) [file ppat.1011319.s005.tif]

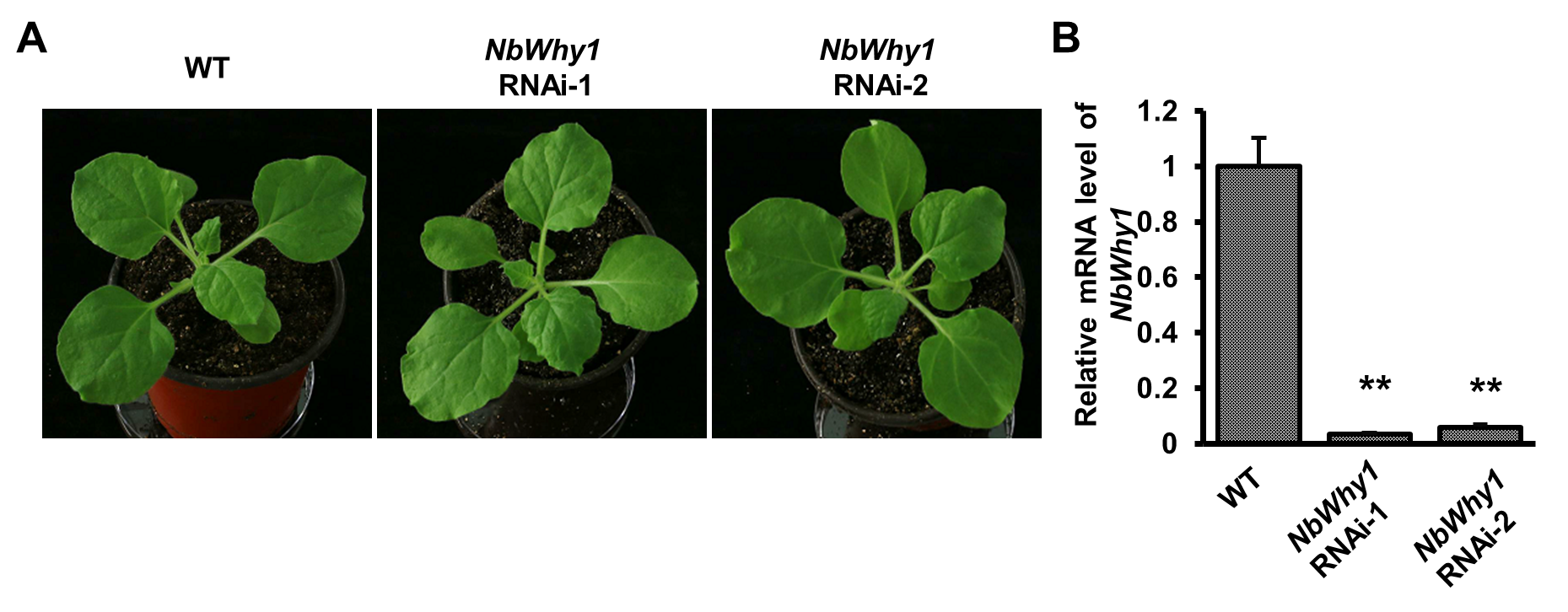

Supplement: S4 Fig — (A) The growth of 5-week-old wild-type (WT) and NbWhy1 RNAi N. benthamiana plants. (B) qRT-PCR analysis of the silencing efficiency of NbWhy1 in plants used in (A). NbGAPDH was used as an internal control. Mean and standard deviation are shown. Double asterisks indicates a significant statistical difference between two treatments at p<0.01 based on Student’s t test. (TIF) [file ppat.1011319.s006.tif]

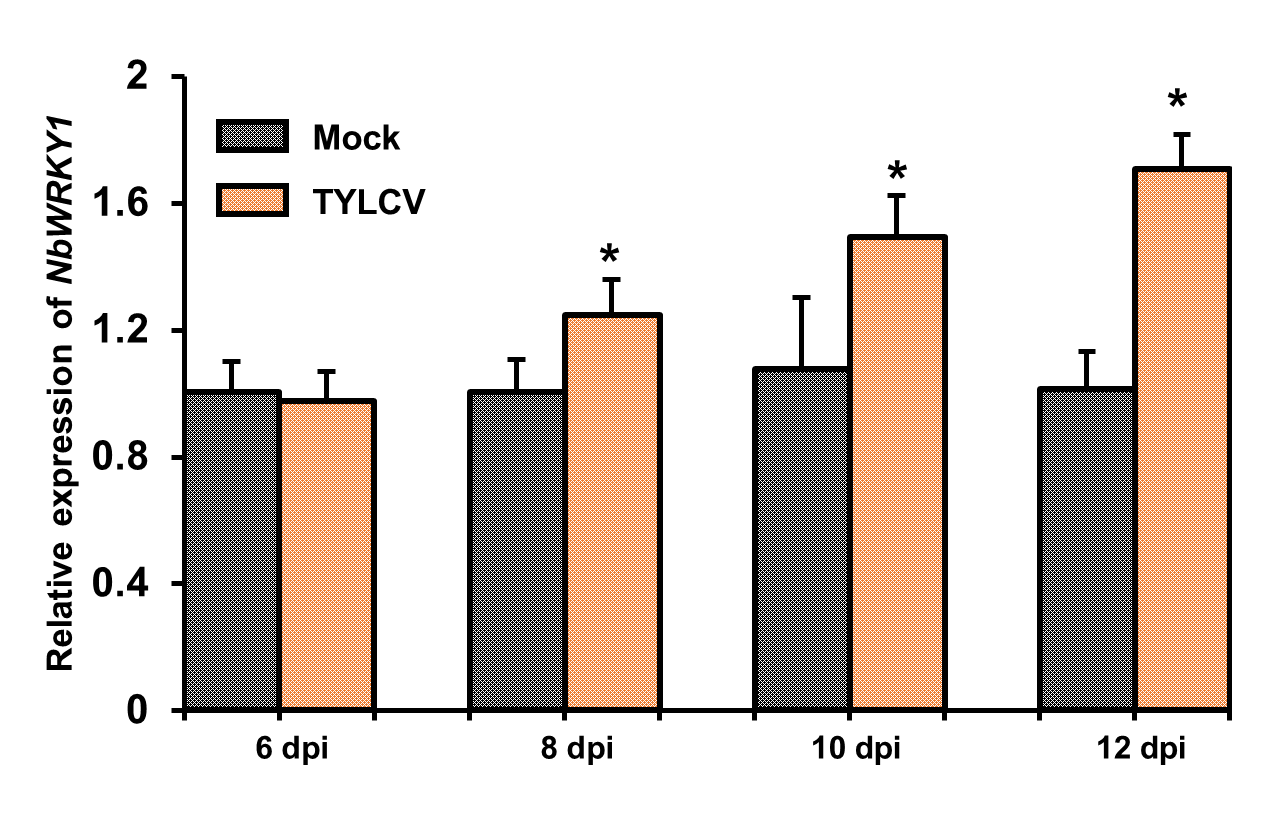

Supplement: S5 Fig — RNA was extracted from the upper non-inoculated plant leaves at various days post inoculation (dpi) as indicated. NbGAPDH was used as an internal control. Asterisks indicate significant statistical differences between two treatments at p<0.05 based on Student’s t test. (TIF) [file ppat.1011319.s007.tif]

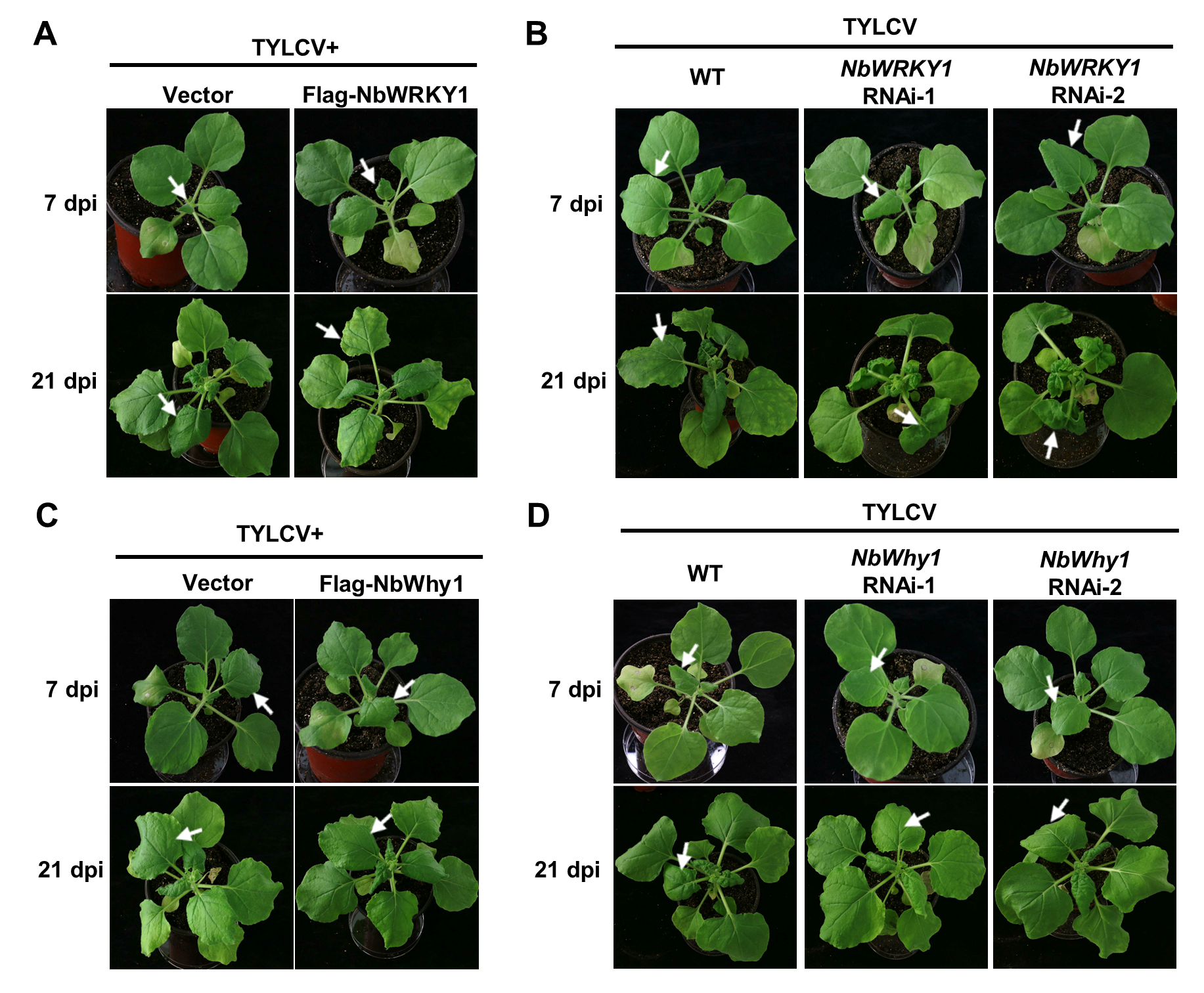

Supplement: S6 Fig — (A) Effect of transient overexpression of NbWRKY1 on the symptoms induced by TYLCV. N. benthamiana plants were inoculated with TYLCV and Flag-NbWRKY1 or TYLCV and pCambia-Flag (the vector control) and symptoms were monitored and recorded at 7 and 21 dpi, respectively. (B) Symptoms of the TYLCV-inoculated wild-type (WT) and NbWRKY1 RNAi N. benthamiana plants at 7 and 21 dpi, respectively. (C) Effect of transient overexpression of NbWhy1 on the symptoms induced by TYLCV. N. benthamiana plants were inoculated with TYLCV and Flag-NbWhy1 or TYLCV and pCambia-Flag (the vector control) and symptoms were monitored and recorded at 7 and 21 dpi, respectively. (D) Symptoms of the TYLCV-inoculated wild-type (WT) and NbWhy1 RNAi N. benthamiana plants at 7 and 21 dpi, respectively. White arrows indicate different severity of the corresponding leaves. (TIF) [file ppat.1011319.s008.tif]

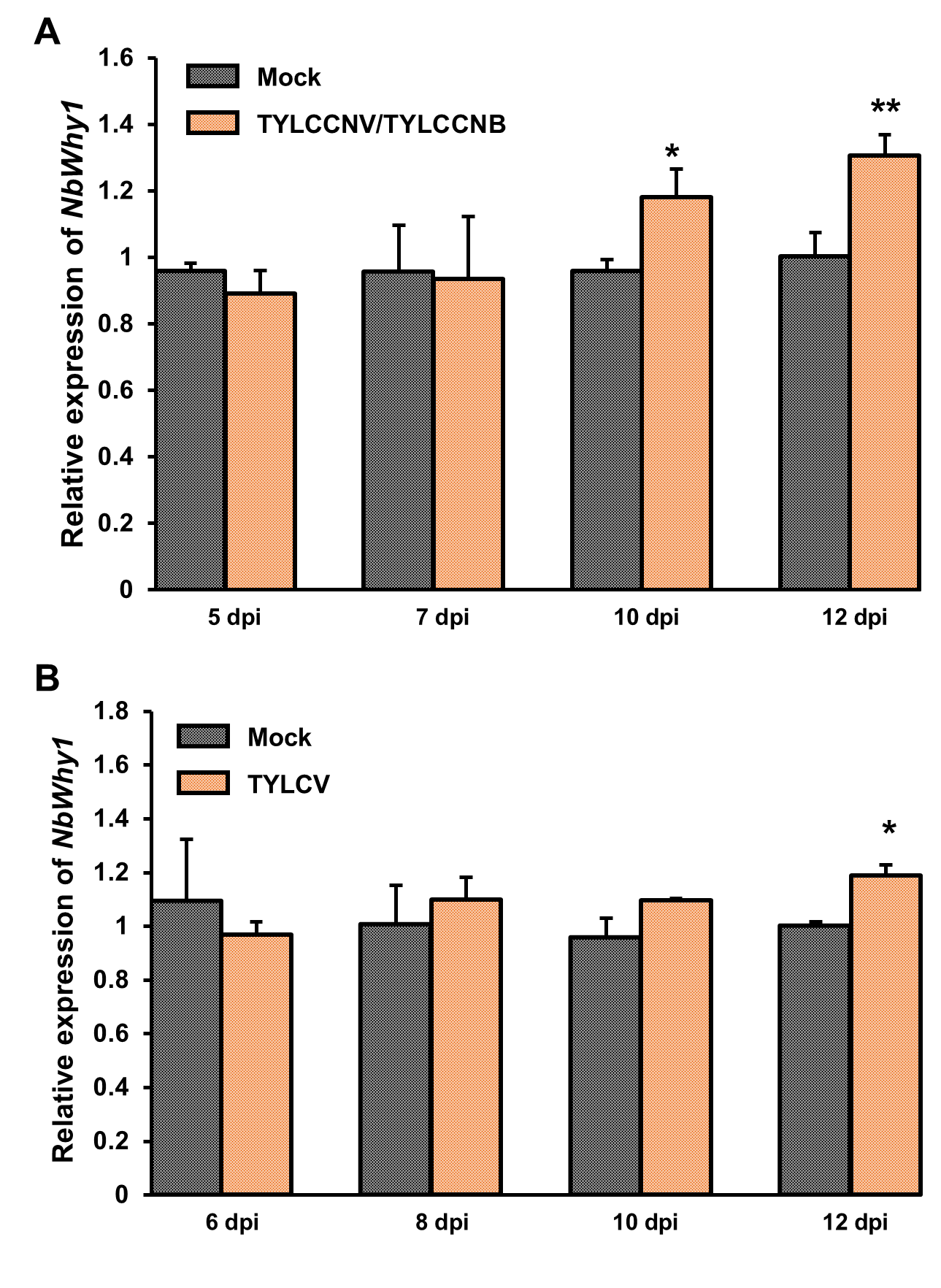

Supplement: S7 Fig — N. benthamiana plants were inoculated with the infectious clone of TYLCCNV/TYLCCNB or TYLCV. Plants inoculated with the empty vector were used as mock controls. RNA was extracted from the upper non-inoculated plant leaves at various days post inoculation (dpi) as indicated. NbGAPDH was used as an internal control. Mean and standard deviation of three independent plants are shown. Double and single asterisks indicate significant statistical differences between two treatments at p<0.01 and p<0.05 based on Student’s t test, respectively. (TIF) [file ppat.1011319.s009.tif]
